# Synphilin-1 modulates alpha-synuclein assembly, release and uptake

**DOI:** 10.1038/s41531-025-01144-3

**Published:** 2025-11-20

**Authors:** Diana F. Lázaro, Triana Amen, Ellen Gerhardt, Chengyuan Song, Ryan Burns, Niels Kruse, Patrícia I. Santos, Dragomir Milovanovic, Günter Höglinger, Brit Mollenhauer, Kelvin C. Luk, Virginia MY- Lee, Tiago F. Outeiro

**Affiliations:** 1 Department of Pathology and Laboratory Medicine, Institute on Aging and Center for Neurodegenerative Disease Research, Perelman School of Medicine at the University of Pennsylvania, Pennsylvania, PA USA; 2 University Medical Center Göttingen, Department of Experimental Neurodegeneration, Center for Biostructural Imaging of Neurodegeneration, Göttingen, Germany; 3 Global Health Institute, École Polytechnique Fédérale de Lausanne, Lausanne, Switzerland; 4 School of Biological Sciences, University of Southampton, Southampton, UK; 5 Department of Neuropathology, University Medical Center Göttingen, Göttingen, Germany; 6 Laboratory of Molecular Neuroscience, German Center for Neurodegenerative Diseases (DZNE), Berlin, Germany; 7 Einstein Center for Neuroscience, Charité-Universitätsmedizin Berlin, Corporate Member of Freie Universität Berlin, Humboldt-Universität Berlin, and Berlin Institute of Health, Berlin, Germany; 8 Institute of Biochemistry, Charité-Universitätsmedizin Berlin, Corporate Member of Freie Universität Berlin, Humboldt-Universität Berlin, and Berlin Institute of Health, Berlin, Germany; 9 Department of Neurology, LMU University Hospital, Ludwig-Maximilians-Universität (LMU), Munich, Germany; 10 German Center for Neurodegenerative Diseases (DZNE), Munich, Germany; 11 Munich Cluster for Systems Neurology (SyNergy), Munich, Germany; 12 Paracelsus-Elena-Klinik, Kassel, Germany; 13 Max Planck Institute for Multidisciplinary Science, Göttingen, Germany; 14 Translational and Clinical Research Institute, Faculty of Medical Sciences, Newcastle University, Framlington Place, Newcastle Upon Tyne, UK; 15 Scientific employee with an honorary contract at Deutsches Zentrum für Neurodegenerative Erkrankungen (DZNE), Göttingen, Germany

**Keywords:** Cell biology, Molecular biology

## Abstract

Alpha-synuclein (aSyn) is an intrinsically disordered protein involved in phase separation and several age-associated neurodegenerative disorders, including Parkinson’s disease. However, its function and pathological role remain elusive. Here, we modeled different aSyn assemblies in living cells by exploiting its interaction with synphilin-1 (Sph1). We developed a model that reports on gel- and solid-like inclusions through coexpression of aSyn and Sph1. Distinct morphological differences emerged between VN-aSyn + aSyn-VC and VN-Sph1 + aSyn-VC assemblies, showing unique antibody recognition, proteinase K resistance, and protein mobilities. The VN-Sph1 + aSyn-VC interaction could be manipulated to alter inclusion size and number. These inclusions also contained lysosomes and AP-1 vesicles, aligning with observations in human brain tissue. Our study offers new insight into aSyn aggregation and release, highlighting the importance of Sph1 and other aSyn-interacting proteins in synucleinopathies, which involve diverse copathologies only now beginning to be understood.

## Introduction

Synphilin-1 (Sph1) was initially identified as an interacting partner of alpha-synuclein (aSyn) in a yeast two-hybrid screen^1^. This interaction was further validated by Fluorescence Resonance Energy Transfer (FRET) in cultured cells^2^, and it was subsequent demonstrated that co-expression of the hydrophobic domain of aSyn (previously known as NAC domain)^3^ with Sph1 resulted in the formation of cytoplasmic inclusions^1,4^. These observations suggest that Sph1 may play an important role in the aggregation process of aSyn, potentially contributing to the formation of pathological inclusions, such as Lewy bodies (LBs) or Lewy neurites (LNs), that are present in the brain of patients affected by Lewy body diseases such as Parkinson’s disease (PD) or Dementia with Lewy bodies (DLB).

Sph1 is expressed in several brain regions, including the *substantia nigra*, hippocampal pyramidal cells, cerebellar Purkinje cells, and glial cells^5–7^, and is also found in peripheral tissues such as the heart and placenta^1^. Despite its widespread expression, the exact physiological function of Sph1 remains unclear. Sph1 has been linked to various cellular processes, including degradation pathways^4^, and synaptic vesicle-binding protein^6,7^. Interestingly, Sph1 has been found to accumulate in LBs and in Glial Cytoplasmatic Inclusions (GCIs)^8,9^, though its specific contribution to the formation of these inclusions has not been fully elucidated. Notably, Sph1 is found within the central core of the LBs, while aSyn is more peripheral^7,8,10^. The Sph1 sequence exhibits several conserved domains, including ankyrin-like repeats and a coiled-coil domain, both of which are known to be facilitate protein-protein interactions,^5^. These structural features suggest that Sph1 may act as a scaffold for the recruitment of various binding partners, including key proteins implicated in PD, such as Parkin, SIAH, LRRK2, and PINK1^4,11–17^. The interaction between Sph1 and these proteins further underscores its potential involvement in the aggregation cascade.

aSyn, is considered to be a central player in the etiology of PD, and its aggregation is a hallmark of the disease^18–21^. This intrinsically disordered protein predominantly localizes at the presynaptic terminals and nucleus^22–24^. Functionally, aSyn is involved in synaptic vesicle trafficking, membrane fusion, and the assembly of SNARE complexes. However, the mechanisms by which aSyn acquires a toxic state remains poorly understood^25–27^. The gain of toxicity associated with aggregated aSyn is believed to result from a combination of factors, including the decline of cellular proteostasis, modifications of the protein through post-translational modifications, and the formation of misfolded oligomers or fibrils^28–31^. While aSyn aggregation is central to PD pathogenesis, the early steps of aggregation are not well characterized. In vitro studies have shown that high concentrations of monomeric aSyn are required for nucleation to occur, and in cell models, seeding or chemical treatments, such as exposure to rotenone or MPTP, are often used to trigger aggregation^32–37^. However, despite these approaches, aSyn does not easily form visible inclusions upon overexpression in either cells or animal models. This challenge highlights the need for new strategies and models to better understand the fundamental principles of the aggregation process of aSyn in the context of neurodegenerative diseases.

Overall, our work highlights Sph1 as a key regulator in aSyn aggregation and suggests that understanding its interaction with aSyn could provide new insights into the mechanisms underlying synucleinopathies.

## Results

### VN-Sph1 + aSyn-VC interaction results in the formation of cytosolic inclusions without impacting cellular homeostasis

**Fig. 1:**
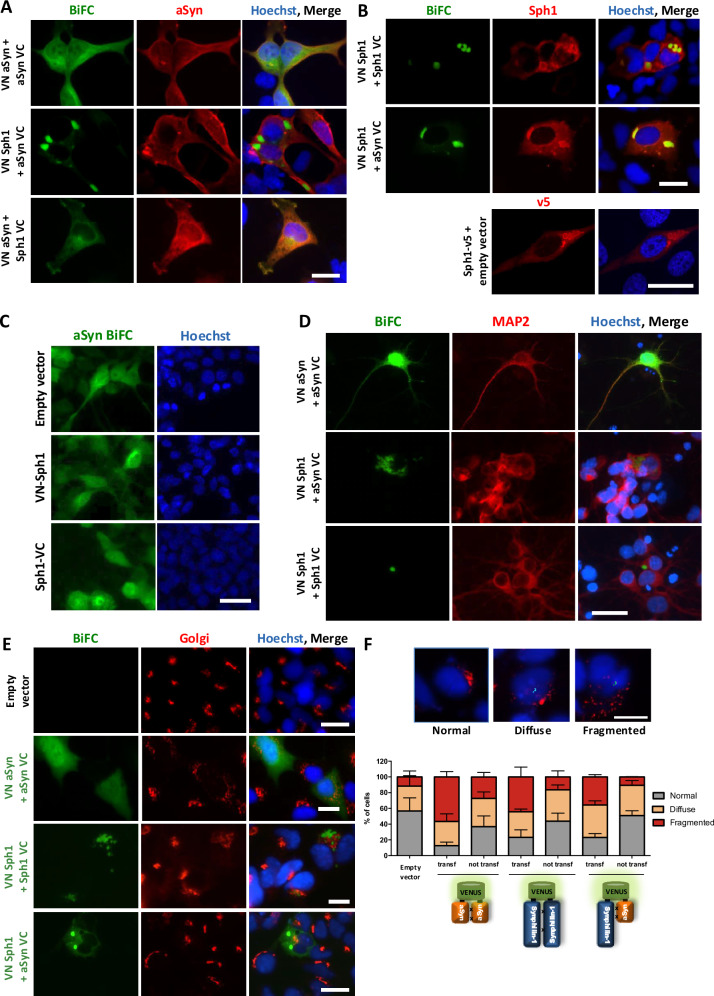
VN-Sph1 + aSyn-VC interaction promotes cytoplasmic inclusions formation in HEK 293 cells and primary cortical neurons and mitigates aSyn-induced Golgi fragmentation. Immunocytochemistry of VN-Sph1 + aSyn-VC inclusions. HEK 293 cells were fixed and stained with either total aSyn (Syn1) (**A**) or anti-Sph1 or anti-V5 antibodies (**B**, upper and lower panel, respectively) (**B**) (scale bar: 30 µm). **C** Competition assay. HEK293 cells were first transfected with aSyn BiFC constructs, followed by re-transfection 24 h with either an empty vector, VN-Sph1 or Sph1. The absence of aSyn inclusions suggests that Sph1 is necessary for initiating aSyn aggregation in cultured cells. **D** Primary cortical neurons exhibit VN-Sph1 + aSyn-VC inclusions. Transfected primary cortical neurons at DIV4 were examined after 48 h (DIV6). Similar inclusions were observed, demonstrating that the model is effective across different cell type (scale bar: 30 µm). **E**, **F** Sph1 rescues the Golgi fragmentation caused by aSyn expression. HEK 293 cells were analyzed 48 h post-transfection, to assess Golgi morphology. Quantification showed that the cells expressing VN-aSyn + aSyn-VCdisplayed significantly increased Golgi fragmentation compared to cells expressing VN-Sph1 + aSyn-VC or Sph1-Sph1. Importantly, Sph1 co-expression reduced Golgi fragmentation by approximately 40%, indicating a potential protective role. Importantly, Golgi fragmentation was not observed in surrounding non-transfected cells, highlighting the specificity of aSyn-induced changes (scale bar: 30 µm). All experiments were performed in triplicate.

To investigate the role of Sph1 on aSyn aggregation, we generated a new Bimolecular Fluorescence Complementation (BiFC) assay for Sph1: VN-Sph1 and Sph1-VC (henceforth referred to as Sph1 BiFC; more information in Materials and Methods section). The two halves of Venus fluorescence protein can be reconstituted upon protein-protein interaction, allowing the direct visualization of dimers/oligomers in living cells. As we previously reported, HEK 293 (HEK) cells co-transfected with VN-aSyn and aSyn-VC (henceforth referred to as aSyn BiFC) typically show a diffuse/homogeneous distribution of the Venus fluorescence signal throughout the cytoplasm and nucleus^38^ (with only a few exceptions^39^). Contrary to what could be expected, aSyn expression in cell models typically does not result in the formation visible aggregates, and this has been a limitation in the field (Fig. 1A, top row). In contrast, cells expressing the VN-Sph1 and Sph1-VC (henceforth referred to as Sph1 BiFC) displayed inclusions with round and elongated/tubular morphology (Fig. 1B, top row). Immunostaining for Sph1 revealed not only inclusions but also soluble Sph1 within the cells, suggesting a correlation between the formation of Sph1 inclusions and protein-protein interaction (Fig. 1B, top row). Consistently, expression of Sph1 from a single plasmid also resulted in the formation of cytoplasmic inclusions (Fig. 1B, lower panel- Sph1-V5), demonstrating that Sph1 can form inclusions independently of the Venus fragment. Furthermore, co-expressing VN-Sph1 + VC alone or VN alone + Sph1-VC did not produce detectable fluorescent signal (supplementary data Fig. 1A). Next, we examined the effects of co-expressing Sph1 together with aSyn. The fluorescent signal confirmed the interaction occurred, but it depended on the orientation of the proteins. The signal was detected in cells co-expressing VN-Sph1 + aSyn-VC already at 24 h (Fig. 1A, supplementary data Fig. 1B), while only a weak signal was detected in cells co-expressing VN-aSyn + Sph1-VC after 48 h (Fig. 1A). Notably, only VN-Sph1 + aSyn-VC resulted in the formation of visible cytoplasmic inclusions (Fig. 1A, B, middle panel).

Next, we assessed the role of phosphorylation on inclusion formation using mutants that attempt to either block (S129A) or mimic (S129D) phosphorylation. No differences were observed in terms of VN-Sph1 + aSyn-VC inclusions (supplementary data Fig. 1C), suggesting that phosphorylation may not affect the interaction between Sph1 and aSyn, nor the formation of the type of cytosolic inclusions formed by the two proteins.

We also asked whether challenging the aSyn BiFC system, by first expressing aSyn BiFC and, after 24 h, introducing VN-Sph1 or Sph1-VC (Fig. 1C), would lead to the formation of cytoplasmic inclusions. In these experiments we did not observe the formation of inclusions, suggesting that the initial interaction is a critical factor for templating aSyn assembly.

Next, we employed this new inclusion-formation model in primary cortical and hippocampal neurons (Fig. 1D and supplementary Figure 1D). As observed in HEK cells, VN-Sph1 + aSyn-VC formed cytosolic inclusions in both cortical and hippocampal neurons. This suggests that the formation of VN-Sph1 + aSyn-VC inclusions occurs in different cell types, including primary neuronal cells, which is a valuable asset for in vitro studies. For the remainder of our study, we continued with the HEK cell model, as this facilitated the types of analyses we aimed for, especially live cell imaging and other cell biological studies in a model that enables the visualization of aSyn inclusions based on the interaction of two proteins found in the LBs without the need for fixation and immunostaining. We also evaluated the toxicity of VN-Sph1 + aSyn-VC expressing using different readouts. We measured the release of adenylate kinase as an indicator of membrane integrity (supplementary data Fig. 2A), and assessed cell viability using two different assays: cell proliferation (supplementary data Fig. 2B) and cell viability (supplementary data Fig. 2C). Overall, we did not detect any signs of cytotoxicity, suggesting that the VN-Sph1 + aSyn-VC species formed were not, per se, detrimental for cellular homeostasis. However, it has been widely reported that aSyn expression may have detrimental implications for different subcellular compartments, such as the Golgi apparatus in the *substantia nigra*^40^. Normally, when the Golgi apparatus is labeled with antibodies like anti-Giantin, it appears as a perinuclear formation, either compact or elongated. In contrast, under stress conditions, the Golgi complex exhibits an increase in discrete Golgi objects and eventually breaks into smaller fragments distributed throughout the cytoplasm^41–43^. Therefore, we also assessed the number of transfected and non-transfected cells with normal, diffuse, and fragmented Golgi (Fig. 1E, F, supplementary data Fig. 2D). Consistent with our previous observation in HEK cells and in neuronal Lund Human Mesencephalic (LUHMES) cells^44,45^, expression of aSyn led to increased Golgi fragmentation (Fig. 1E, F and supplementary data Fig. 2D, red bars). This phenotype was associated with aSyn expression, as non-transfected cells did not exhibit the Golgi alterations. Importantly, Sph1 counterbalanced the effects of aSyn, as co-expression of both proteins reduced Golgi fragmentation by approximately 20% in comparison to VN-aSyn + aSyn-VC (Fig. 1E, F). To further validate our observations, we synchronized the cultures by inducing cell cycle arrest at the G1/S boundary using a double-thymidine S phase block^46^. Golgi fragmentation is known to occur during various cellular processes, including cell division, cell death, migration, differentiation, and polarization, and can be either reversible or irreversible. To ensure that the observed Golgi fragmentation was specifically associated with aSyn expression, we synchronized the cell cycle and restricted our analysis to cells in the G1/S phase. After removing the S phase block through thymidine washout, and expressing aSyn and Sph1 (additional details in the Materials and Methods section) cells were fixed 12 h post-thymidine removal, and the morphology of the Golgi apparatus was assessed as before. Overall, we observed no differences between untreated cells (not synchronized) and cells treated with thymidine (at the G1/S phase), indicating that the effects were indeed attributable to aSyn expression rather than differences in the cell cycle stage of the cells (supplementary data Fig. 2E). We then analyzed the area occupied by the Golgi apparatus using a semi-automated system. The rationale was that fragmented Golgi would occupy a larger area, as the smaller fragments are dispersed throughout the cytoplasm, thus covering a greater surface area. Due to the diverse shapes that the Golgi can adopt, we used a plugin from ImageJ (Hull and Circle) to extract some shape descriptors such as the convex area (supplementary data Fig. 2F). We selected the convex area data as the most accurate approximation of the area occupied by the Golgi. As illustrated by the frequency distribution, cells expressing aSyn BiFC exhibit a lower percentage of Golgi area for smaller sizes, but increases with the larger areas (supplementary data Fig. 2F, orange bars). In contrast, VN-Sph1 + aSyn-VC displayed a frequency distribution more similar to that observed with the control cells. This semi-automatic quantification corroborated our earlier quantifications, indicating that aSyn BiFC occupies a larger area compared to either control cells or VN-Sph1 + aSyn-VC. Overall, these results suggest that the interaction between both proteins is beneficial, reducing proteostasis stress.

**Fig. 2:**
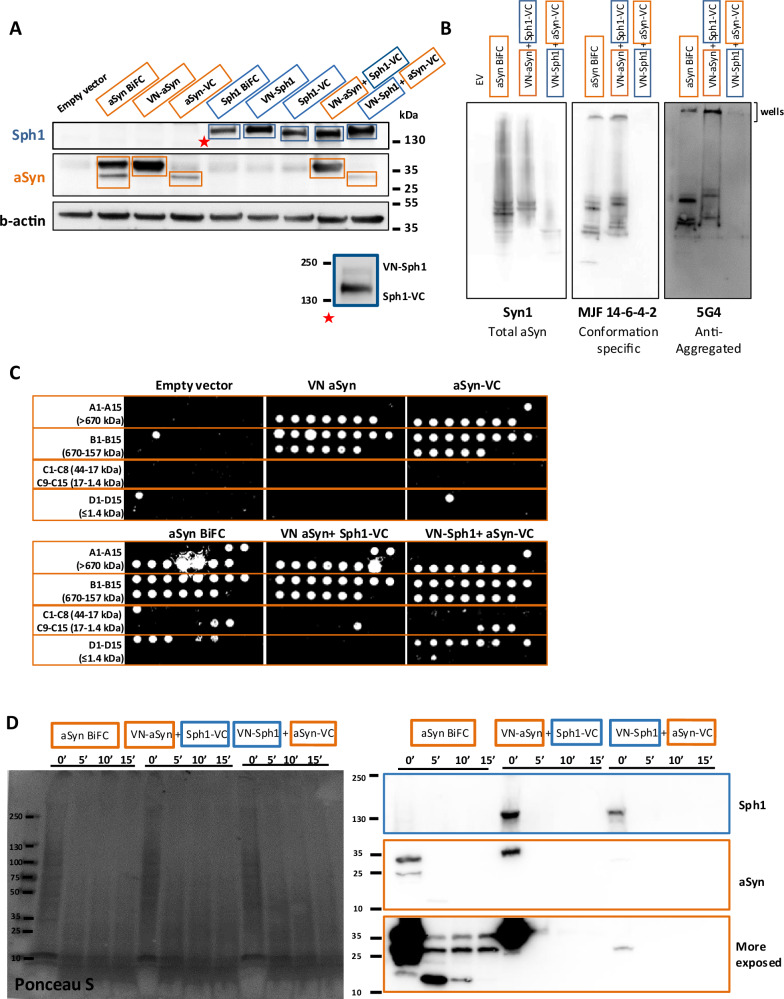
Biochemical characterization of VN-Sph1 + aSyn-VC inclusions. **A** Expression levels of aSyn and Sph1. Immunoblot analyses was performed to determine the expression levels of aSyn (orange box) and Sph1 (blue box). A separate longer exposure (blue square) for Sph1 BiFC reveals the separation of both VN-Sph1 and Sph1-VC, indicating distinct expression forms of the protein fusion constructs. All the above experiments were performed at least three times. **B**, **C** Molecular properties of VN-Sph1 + aSyn-VC inclusions. Under non-denaturing conditions, aSyn oligomeric smears were detected (**B**, n = 2). HPLC/dot blot assay, no significant shifts in molecular weight of aSyn were detected, indicating that VN-Sph1 + aSyn-VC inclusions may represent a phase transition or/and an amorphous protein state rather than defined higher-order structures (**C**, n = 2). **D** VN-Sph1 + aSyn-VC inclusions are less resistant to PK than VN-aSyn + aSyn-VC. Cell lysates were treated with PK for 0, 5, and 15 min. n = 3.

### VN-Sph1 + aSyn-VC assemblies have limited aggregation propensity and altered structural stability

To investigate whether these effects were related to differences in aSyn levels, we performed immunoblot analysis (Fig. 2A). We found that, overall, the levels of aSyn-VC were lower than VN-aSyn. However, it is important to note that these observations are not indicative of the degradation of aSyn-VC, as our group and others have reported similar findings^39,44,47–51^. Although it may appear unexpected, we consider this distinction as biologically significant, given that protein levels in cells are affected by a multitude of factors, thereby contributing to biological diversity. Additionally, to account for the molecular weight differences between aSyn and Sph1, we ran separate gels for Sph1 BiFC to resolve VN-Sph1 and Sph1-VC (Fig. 2A, blue rectangle panel).

To understand the biogenesis of VN-Sph1 + aSyn-VC inclusions, we employed several biochemical assays to measure the protein aggregation state and stability. We started by analyzing the properties of VN-Sph1 + aSyn-VC complexes in native conditions using different aSyn antibodies. Overall, we did not detect any band using the MJF 14-6-4-2 antibody (a conformation-specific antibody that detects aggregated aSyn), and observed only faint bands for Syn1 (which detects total aSyn) and 5G4 antibody (recognizing aggregated aSyn), with an upper band located in the well region of the gel for VN-Sph1 + aSyn-VC (Fig. 2B). As mentioned earlier, aSyn-VC is less abundant when compared to VN-aSyn (Fig. 2A). Therefore, this suggests that either the amount of aSyn present in the sample is below the detection range of the assay or aSyn is not fibrillized. To further characterize the nature of these inclusions, we performed size exclusion chromatography by high-performance liquid chromatography (SEC-HPLC), followed by dot blot analysis, detecting aSyn using the Syn1 antibody. Co-expression of the proteins- either VN-aSyn + Sph1-VC or VN-Sph1 + aSyn-VC- did not significantly alter the high molecular weight species of aSyn species compared with the controls (VN- or -VC) (Fig. 2C). Instead, VN-Sph1 + aSyn-VC formed small assemblies (Fig. 2C).

Next, we assessed the structural stability of these assemblies by treating the samples with PK. We found that the VN-aSyn + aSyn-VC interaction is resistant to PK digestion, resulting in lower fragments around 15 kDa. The PK resistance patterns for both VN-aSyn + Sph1-VC and VN-Sph1 + aSyn-VC were similar, and collectively, they were less-resistant to PK digestion compared to aSyn BiFC (Fig. 2D). Collectivity, these data showed slight changes of aSyn at the molecular level, challenging our initial premise that VN-Sph1 + aSyn-VC may forms amorphous inclusions rather than aggregated clusters.

### VN-Sph1 + aSyn-VC inclusions are hydrogel-like phase-separated assemblies

**Fig. 3:**
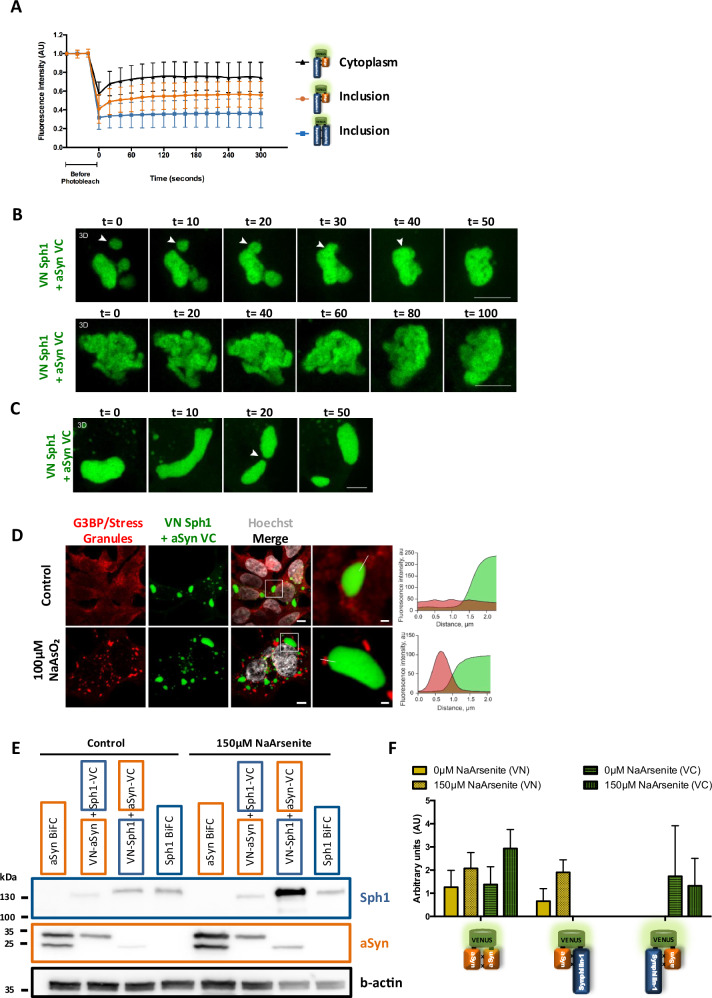
Gel-like properties of VN-Sph1 + aSyn-VC assemblies. **A** VN-Sph1 + aSyn-VC inclusions exhibit reduced mobility. FRAP was performed to assess the mobility of the inclusions. VN-Sph1 + aSyn-VC inclusions displayed slower recovery rates compared to VN-aSyn + aSyn-VC inclusions, suggesting that VN-Sph1 + aSyn-VC adopts a more gel-like state with reduced mobility. **B**, **C** Dynamics of VN-Sph1 + aSyn-VC inclusions. Live-cell imaging revealed that small or single VN-Sph1 + aSyn-VC inclusions fused into a larger aggregate over time, while tubular inclusions exhibited reduced mobile (**B**). Upon cycloheximide treatment, the inclusions became more fluid, facilitating fusion and fission events. However, after 24 h of treatment, inclusions were not fully dissolved (scale bar: 5 µm) (**C**). **D** Stress granules do not co-localize with VN-Sph1 + aSyn-VC. Representative confocal images of cells expressing VN-Sph1 + aSyn-VC under control conditions or arsenite treatment (100 µM for 1 h), were stained with G3BP antibody (a marker of stress granules). Fluorescence intensity profiles (shown along the white line in inset images) indicated no co-localization between stress granules and VN-Sph1 + aSyn-VC inclusions. Scale bar of overall image: 5 µm and crop image: 1 µm. **E**, **F** WB quantification. Protein levels were quantified, and the relative expression levels of the proteins were plotted from three independent experiments.

Several intrinsically disordered proteins, such as TAR DNA-binding protein 43 (TPD 43), Fused in sarcoma (FUS) and Tau, are known to undergo phase separation processes^52^. Recent studies have shown that aSyn can form condensates through phase separation, and that this liquid–liquid phase separation occurs prior to aggregation into insoluble species^53^. Therefore, to investigate whether these inclusions might represent a phase-separated liquid droplet, we used Fluorescence Recovery After Photobleaching (FRAP) to assess the protein state and mobility in living cells. We analyzed the recovery data for aSyn-aSyn, VN-Sph1 + aSyn-VC, and Sph1-Sph1 across cytoplasmic regions and/or inclusions over a 5 minutes period (Fig. 3A and supplementary data 3A). The fastest dynamics were observed for VN-aSyn + aSyn-VC (data not shown due to complete fluorescence recovery being outside of the photodetector sensitivity limit), indicating the presence of a soluble, freely diffusible state of VN-aSyn + aSyn-VC species. The FRAP recovery curves for VN-Sph1 + aSyn-VC inclusions (Fig. 3A, orange circle) revealed a notably slower half-life recovery (supplementary data Fig. 3B orange graph) compared to the diffuse VN-Sph1 + aSyn-VC signal regions (Fig. 3A, black triangle; supplementary data Fig. 3B black graph). These differences suggest a distinct organizational structure within the inclusions. In contrast, Sph1 BiFC assemblies remained largely immobile (Fig. 3A, blue square; supplementary data Fig. 3B blue graph), exhibiting minimal signal recovery post-photobleaching. These results imply that the interaction between Sph1 and aSyn significantly alters the properties of both proteins, with VN-Sph1 + aSyn-VC inclusions likely adopting a specific conformation characteristic of a gel-like state.

Next, we performed live cell imaging to determine the dynamics of VN-Sph1 + aSyn-VC. Generally, liquid-like condensates can grow through two mechanisms: Ostwald ripening (where diffusion-limited molecules movement from small to large droplets) or coalescence (where small droplets come into close proximity, touch, and then fuse into a larger droplet)^54^. In our model, we observed two distinct morphologies of VN-Sph1 + aSyn-VC inclusions: round and/or elongate/tubular. Notably, VN-Sph1 + aSyn-VC inclusions, whether multiple small ones or single large one, consistently fused to form larger aggregates (Fig. 3B, upper panel). The elongated/tubular inclusions displayed reduced mobility (Fig. 3B, lower panel and Supplementary movie 3C upper and lower), and did not exhibit significantly change or fusion within the same time scales. Strikingly, treatment with cycloheximide increased the overall fluidity of the inclusions, enabling them to break apart. However, this treatment was insufficient to completely dissolve VN-Sph1 + aSyn-VC inclusions from the cytoplasm, even after 24 hours of treatment (Fig. 3C and Supplementary movie 3C). Next, we examined the effect of oxidative stress by inducing the production of reactive oxygen species through sodium arsenite treatment. Although this treatment led to the formation of stress granules, we did not observe any colocalization or significant alteration in VN-Sph1 + aSyn-VC inclusions (Fig. 3D). Overall, we observed a trend of increased expression for VN-aSyn + aSyn-VC and VN-aSyn + Sph1-VC, while VN-Sph1 + aSyn-VC showed a decrease. However, due to the variation among the replicates, none of these results are statistically significant (Fig. 3E, F). These findings suggest that the VN-Sph1 + aSyn-VC complex adopt a conformation resembling a gel-like state, with sufficient mobility that allows for fusion and the formation of larger aggregates.

### Lysosomes and AP-1 localize within VN-Sph1 + aSyn-VC assemblies

**Fig. 4:**
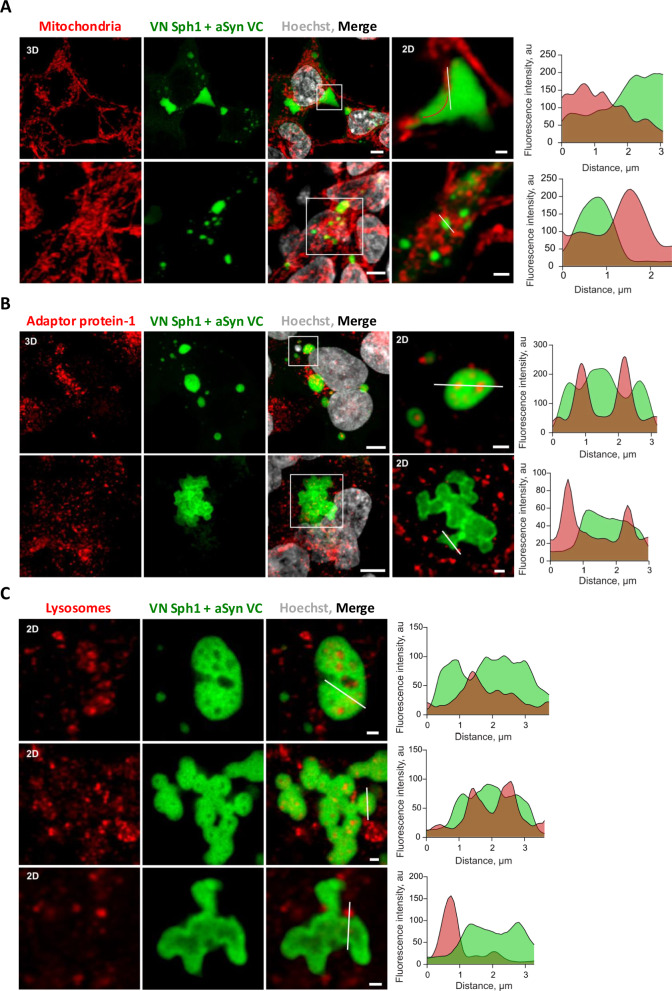
Lysosomes and AP-1 are trapped within VN-Sph1 + aSyn-VC inclusions. Confocal imaging was performed on HEK293T cells overexpressing VN-Sph1 + aSyn-VC to visualize several cellular makers. Mitochondria (labeled with TOM20) were observed to localize proximal or even passing through VN-Sph1 + aSyn-VC inclusions (red line) (**A**). Notably, lysosomes (label with LAMP1) and Adaptor protein-1 (AP-1) were found to be trapped within VN-Sph1 + aSyn-VC inclusion (**B**, **C**). Fluorescence intensity profiles (white line on the inset)- the graphs displayed on the right side of each image panel further emphasize the co-localization of these two markers within the inclusions. Scale bar of overall image: 5 µm and crop image: 1 µm.

During our imaging acquisitions, we frequently observed the presence of dark spots embedded within the VN-Sph1 + aSyn-VC assemblies. As several studies have shown, during aSyn aggregation, particularly in the pathological context, these aggregates often incorporate organelles such as mitochondria and lysosomes, as well as lipids^55^^,^^56^. To investigate whether VN-Sph1 + aSyn-VC inclusions were associated with specific cellular organelles, we monitored various membrane-bound and non-membrane-bound compartments during the formation of VN-Sph1 + aSyn-VC inclusion (Fig. 4 and supplementary data Fig. 4). Interestingly, the inclusions did not co-localize with any of the examined organelles, including lipid droplets, peroxisomes, mitochondria, endoplasmic reticulum, and P bodies (supplementary data Fig. 4). We observed only a slight reduction in P bodies, while some mitochondria were found in close proximity to the inclusions, occasionally passing through them (Fig. 4A). Otherwise, no significant alterations in organelle morphology were detected, except for the fragmentation of the Golgi, as we previously described.

We also observed that VN-Sph1 + aSyn-VC inclusions were frequently localized proximal to the plasma membrane side of the Golgi apparatus (trans-Golgi), which serves as a crucial hub for membrane trafficking that directs vesicles towards lysosome formation or secretion. Notably, only post-Golgi compartments, and not any other cellular compartment, were found within the VN-Sph1 + aSyn-VC inclusions (Fig. 4B, C). Vesicles derived from the trans-Golgi network (TGN), marked with gamma-adaptin (AP-1, Adaptin γ), and lysosomes (marked with LAMP1) were found inside the inclusions, although not within the tubular inclusions. This indicates that VN-Sph1 + aSyn-VC assembly may disrupt normal membrane trafficking originating from the TGN. These findings suggest that the persistent presence of these inclusions may compromise cellular degradation system, such as the ubiquitin–proteasome system and/or autophagy–lysosomal pathway, as well as an impairment at the level of the TGN.

### Sph1 plays an important role on aSyn aggregation

**Fig. 5:**
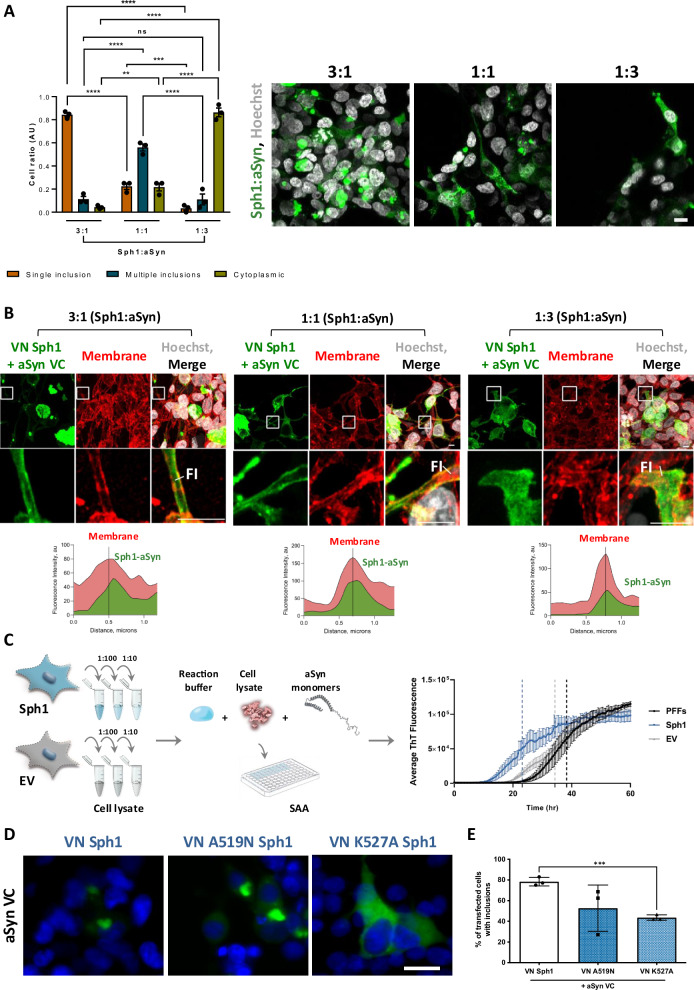
Modulation of VN-Sph1 + aSyn-VC inclusion formation. **A** Different levels of expression of aSyn and Sph1 alter the inclusion phenotype. High Sph1 expression preferentially leads to the formation of tubular inclusion, whereas increased aSyn levels promote the formation of smaller, multiple inclusions. **B** VN-Sph1 + aSyn-VC localize at the membrane. Regardless of the relative expression levels of Sph1 and aSyn, clusters of VN-Sph1 + aSyn-VC are observed within the membrane. This observation becomes more noticeable when stable expression of Sph1 and aSyn is promoted. Scale bar: 10 µm. **C** Sph1 seeded monomeric aSyn into fibrils faster than monomeric aSyn. Mixing Sph1 cell lysate with monomeric aSyn promoted a faster aggregation than either monomeric aSyn or cell lysate. **D**, **E** Disruption of VN-Sph1 + aSyn-VC interaction reduces inclusion formation. Mutation in the Sph1 region, particularly at position 527, which is proposed to interact with aSyn, lead to a significant reduction in inclusions formation. These results indicate that the VN-Sph1 + aSyn-VC interaction is critical for the assembly of these inclusions. Scale bar: 30 µm.

The interaction between Sph1 and aSyn is important for the formation of intracellular inclusions, although the contribution of each protein to this process remains unclear. To explore whether Sph1 drives the process, we expressed Sph1 and aSyn at different ratios. Our results demonstrated that Sph1 expression predominantly led to the formation of a single inclusion, while aSyn resulted in a more cytoplasmic distribution (Fig. 5A), in line with aSyn having a high diffusion rates and acting as a fluidizer at the synapse as well^57,58^. These findings suggest that Sph1 plays a primary role in driving the assembly process, highlighting the importance of protein-protein interactions and the orientation of the proteins as essential driving force. Furthermore, and independent of the expression ratio, we observed VN-Sph1 + aSyn-VC BiFC signal proximal to the plasma membrane (Fig. 5B and supplementary data Fig. 5A). These observations are not a surprise, as it is known that aSyn interacts with lipid membranes; however, many models have failed to replicate this aspect. To support our observations, we designed a plasmid encoding for a fusion between Sph1 and aSyn, to ensure equivalent expression levels of both proteins. As anticipated, we confirmed the presence of VN-Sph1 + aSyn-VC at the membrane (see supplementary data Fig. 5A, VN-Sph1 + aSyn-VC panel), reinforcing the role of Sph1 in modulating membrane localization of VN-Sph1 + aSyn-VC.

The aSyn seed amplification assay (SAA) is an essential tool for detecting minimal levels of aggregated aSyn. Given that pathological aSyn aggregates are thought to associate with disease progression, even at low concentrations, we used SAA to quantitatively assess the aggregation propensity of aSyn in the presence of Sph1, providing insights into how Sph1 may act as a molecular trigger for aSyn aggregation. Diluted lysates from cells expressing GFP-Sph1 or GFP (empty vector- EV) were combined with monomeric aSyn and incubated for up to 60 h (Fig. 5C). A lag phase of 8–10 h was observed, followed by a rapid increase in fluorescence (Fig. 5C). The Sph1 samples seeded monomeric aSyn into fibrils faster, as evidenced by a shorter lag phase in ThT fluorescence (EC50 = 23.13) compared with PFFs (EC50 = 38.27) and empty vector (EV; EC50 = 34.31) (Fig. 5C). The maximum fluorescence intensity (Imax) for all the samples were similar, but the ThT area under the curve (AUC) of the SAA aggregation curve was different. We defined AUC since it summarizes all the kinetic features of the aggregation reaction, including the speed and extent of aggregation. The SAA AUC from PFFs was lower compared to Sph1 (supplementary data Fig. 5E), supporting not only that the environment context influence aSyn aggregation, but Sph1 accelerates aSyn aggregation. To validate the important role of Sph1 on aSyn aggregation, we hypothesized that disrupting the interaction between these two proteins would reduce inclusion formation. To test this hypothesis, we strategically mutated specific Sph1 residues known to be important for VN-Sph1 + aSyn-VC interaction^11^. We specifically targeted residues at positions 519 and 527 based on the published dissociation constants (K_D_), which indicates the strength of the interaction between a molecule and its binding partner, and because these two residues were relatively well separated from each other^11^. The two mutants led to an overall decrease in the number of inclusions within the cells (Fig. 5D, E), specifically for K527A Sph1 mutant, with 40% of the cells displaying less inclusions compared to those expressing WT Sph1 (Fig. 5E). Overall, these results provide robust evidence for the critical role of the VN-Sph1 + aSyn-VC interaction in driving aSyn assembly and inclusion formation.

Protein aggregation is a complex and dynamic process involving the misfolding, accumulation, and aggregation of proteins over time. This process can be influenced by multiple factors, including cellular stress, protein expression levels, and molecular chaperones. As we and others have previously reported, expression of Heat shock proteins (Hsp) 70 or Hsp27 can reduce aSyn toxicity and aggregation in both cell and animal models^38,59–61^. Therefore, we investigated whether Hsp expression could modulate the interaction between Sph1 and aSyn in our new cell model. Expression of Hsp70 significantly reduced the percentage of cells displaying inclusions, with approximately 70% of the cells showing no inclusions (Supplementary data Fig. 5B). This reduction was further corroborated by a decrease in the area of VN-Sph1 + aSyn-VC inclusions (Supplementary data Fig. 5C). Notably, this effect appeared to be specific to the VN-Sph1 + aSyn-VC interaction, as no effect was observed for Sph1 BiFC inclusions (Supplementary data Fig. 5C). In fact, overexpression of Hsp27 or Hsp70 resulted in an increase in the size of Sph1 inclusions, suggesting that these chaperones may selectively modulate the interaction dynamics between Sph1 and aSyn. Importantly, these changes were not associated with alterations in the total protein levels of Sph1 or aSyn, indicating that the observed effects were not due to differences in protein expression but rather reflect changes in aggregation behavior (Supplementary data Fig. 5D). This implies that Hsp70 and Hsp27 influence Sph1 aggregation through distinct mechanisms, potentially altering the properties of the VN-Sph1 + aSyn-VC complex in different ways.

These findings suggest that different Hsps can selectively modulate the interaction between VN-Sph1 + aSyn-VC, influencing both the aggregation dynamics and inclusion morphology. Our results highlight distinct, chaperone-specific mechanisms by which Hsps impact protein aggregation.

### Sph1 modulates aSyn release and uptake

**Fig. 6:**
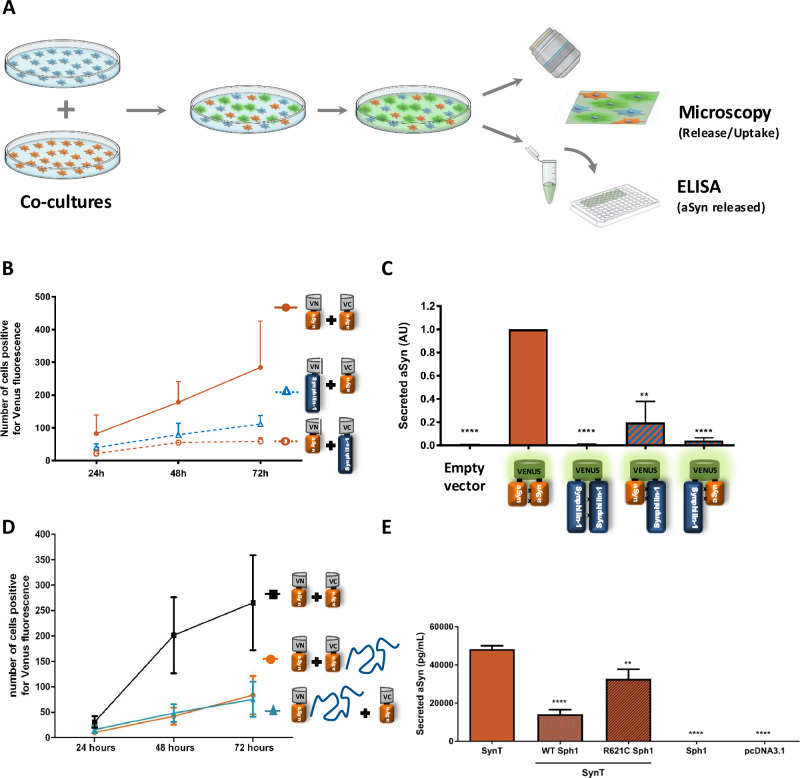
Sph1 modulates aSyn secretion and uptake. **A** Experimental design. Cells were independently transfected with either the VN- or the -VC plasmids. The following day, equal number of cells were mixed and left in co-culture for up to 72 h. If aSyn or Sph1 are released and subsequently taken up by neighboring cells, fluorescent cells should be detected. **B** Quantification of mix BiFC-positive cells in co-cultures. Cells transfected with either VN-aSyn, aSyn-VC, VN-Sph1 or Sph1-VC constructs were co-cultured with an equal number of cells for up to 72 h. The reconstitution of the Venus fluorescence was lower when Sph1 expressing cells were co-culture with aSyn, suggesting a reduced release or uptake. n = 2. **C** Quantification of aSyn in culture media. Media from the co-cultured cells described in (**A**) were collected, and aSyn levels were measured. The presence of Sph1 resulted in a significant reduction in aSyn secretion into the media. ****P < 0.0001 by paired, two-tailed Student’s *t*-test. n = 3. **D** Quantification of aSyn BiFC-positive cells from co-cultures. Cells transfected with VN-aSyn or aSyn-VC were co-cultured with their complementary pairs, aSyn-VC + Sph1 or VN-aSyn + Sph1 for 72 h. The presence of Sph1 reduced the number of aSyn BiFC-positive cells. n = 3. **E** Secretion of aSyn using the aggregation model. Media from H4 cells were collected to measure aSyn secretion. Both WT and mutant Sph1 reduced the amount of aSyn secreted into the media ****P < 0.0001 by paired, two-tailed Student’s *t*-test. n = 3.

To investigate the role of Sph1 on aSyn release and uptake, we conducted co-culture experiments. In these experiments, one construct was expressed in a set of donor cells, while the complementary construct was expressed in recipient cells. This setup enabled us to investigate whether proteins released into the media by donor cells were taken up by recipient cells, resulting in fluorophore reconstitution and detectable fluorescence^47,62^. We quantified the number of fluorescent cells as an indicator of spreading (protein release and uptake), and we also measured the amount of aSyn present in the media by ELISA (Fig. 6A). Overall, we observed a lower number of fluorescent cells when Sph1 was present, suggesting that Sph1 may reduce aSyn uptake (Fig. 6B). However, the numbers for VN-aSyn cells co-cultured with Sph1-VC may be underestimated due to potential inefficiencies in the interaction between Sph1 and aSyn as previously reported. To further clarify this, we collected the culture medium for aSyn quantification using MesoScale analysis^39^. The results supported our initial findings, showing that the presence of Sph1 correlated with reduced aSyn release into the media (Fig. 6C). Given our previously observed differences in aSyn-VC and VN-aSyn expression levels, we conducted an additional round of co-culture experiments with both aSyn BiFC and Sph1 expression. However, this time we performed co-cultures of aSyn-VC + Sph1 with VN-aSyn, or vice versa. Again, we detected fewer fluorescent cells in the presence of Sph1 compared to VN-aSyn co-cultured with aSyn-VC (Fig. 6D), a result independent of any membrane impairment (Supplementary Fig. 5F). Finally, we employed a different aggregation model, SynT + Sph1^39,63^, to visualize aSyn inclusions in fixed cells (Supplementary Fig. 5G). Co-expression of SynT with either WT or mutant Sph1 (R621C, associated with sporadic cases of PD) showed no significant change in inclusion numbers, though Sph1 consistently reduced aSyn levels in the medium.

Collectively, our findings demonstrate that Sph1 decreases extracellular aSyn levels, indicating a role in modulating aSyn release. Additionally, these results suggest that Sph1 may influence aSyn uptake by neighboring cells, pointing to a previously unrecognized function of Sph1 in regulating aSyn trafficking dynamics. This regulatory role of Sph1 could be critical to understanding the mechanisms underlying synucleinopathies.

## Discussion

The mechanisms involved in the assembly of aSyn into ordered filaments, which contributes to the formation of intracellular inclusions in PD and related Synucleinopathies, remains a topic of intense research. Despite the existence of a variety of in vitro and in vivo models for studying aSyn aggregation, these models often fail to mimic the complexity of the aggregation process, for reasons we still do not fully understand, Typically, aggregation has been induced through the use of chemical stressors, inhibitors of the activity of quality control components (e.g. the proteasome system or autophagy), or by external stimuli, like the addition of a fibrillar forms of recombinant aSyn. However, such methods often fall short in replicating key aspects of aSyn aggregation, suggesting that additional factors, such as specific protein interactions, likely influence the aggregation process. Our study introduces a novel, cell-based model for investigating the mechanisms of aSyn aggregation, and identifies Sph1 as a pivotal modulator of this process through direct protein-protein interactions. This work sheds light into the molecular determinants underlying the spatial and structural organization of aSyn-containing inclusions, offering new insights into their formation, dynamics, and cellular consequences.

We demonstrate that Sph1 expression impacts both the size and number of VN-Sph1 + aSyn-VC inclusions, likely by acting as a scaffold that recruits aSyn through its coiled-coil domain or by altering the expression levels of these proteins. Notably, the dynamic nature of these inclusions, which undergo fusion and fission, suggests a regulated process that can be tuned by altering Sph1 or aSyn expression levels or by disrupting their interaction. Based on our data, we found no evidence to suggest that the interaction between Sph1 and aSyn is transient. In fact, some of the inclusions observed were highly stable and immobile. Although these structures occasionally fragmented, we did not observe complete clearance of VN-Sph1 + aSyn-VC inclusions throughout the duration of our experiments. This dynamic behavior, coupled with the tendency of inclusions to fuse, is reminiscent of liquid–liquid phase separation (LLPS), a biophysical process increasingly recognized as a key mechanism driving the formation of membraneless cellular compartments. Recent studies have demonstrated that aSyn itself can undergo LLPS under physiological and pathological conditions, forming condensates that exhibit liquid-like properties, which can progressively mature into more gel-like or solid states over time^53,64,65^. The observed fusion and fission events, as well as the stability of certain inclusions, suggest that the Sph1–aSyn assemblies may similarly originate from phase-separated condensates. This model of LLPS-driven inclusion formation would be consistent with the scaffold-client paradigm, wherein Sph1 could act as a scaffold protein, nucleating the recruitment of the more dynamic and intrinsically disordered aSyn, ultimately facilitating the formation and maturation of cytoplasmic inclusions. Furthermore, the presence of tubular-like structures suggests that these assemblies can matured into more ordered, anisotropic assemblies. Functionally, these tubular inclusions may serve as sequestration sites that modulate aSyn toxicity by compartmentalizing misfolded or aggregation-prone species, thus initially acting as protective scaffolds. However, we can not discard that the persistence of such structures could impair intracellular trafficking and protein homeostasis, contributing to neuronal dysfunction. For example, TDP-43 is an intrinsically disordered RNA-binding protein that plays a critical role in RNA metabolism and gene regulation. Due to its low-complexity domains, TDP-43 undergoes LLPS to form dynamic ribonucleoprotein condensates under physiological conditions^66,67^. However, pathological perturbations can drive these condensates to mature into more solid-like, fibrillar assemblies, resulting in the characteristic skein-like inclusions^68^ observed in neurodegenerative diseases such as amyotrophic lateral sclerosis and frontotemporal dementia^69^. Additionally, the specificity of our models allowed us to gain deeper insight into this interaction. Our data indicate that the effects we observed are driven by the orientation of Sph1 and aSyn, as demonstrated by the VN-aSyn + Sph1-VC condition. In this configuration, not only was an additional 24 h required for a detectable signal to appear, but the resulting inclusions were markedly fewer or, in some instances, absent compared to the VN-Sph1 + aSyn-VC condition (Fig. 1A). These findings suggest that the more flexible structure of aSyn, particularly when its N-terminus remains free, makes the interaction with Sph1 more favorable.

To corroborate these observations, we demonstrated that disrupting this interaction significantly reduces inclusion formation (Fig. 5C), indicating that beyond protein orientation, the coiled-coil domains of Sph1 are also critical for inclusion assembly. Our results provide cellular evidence supporting the essential role of the coiled-coil domain in the recruitment of aSyn and the subsequent formation of cytoplasmic inclusions^4–6^. Taken together, these findings suggest that the interaction between Sph1 and aSyn, modulated by this structural motif, represents a key determinant of the aggregation process, consistent with previous studies and further underscores the importance of this domain. To address the limitations inherent to this model and to further validate our findings, we employed complementary approaches, including the Seeding Amplification Assay. Consistently, in this assay, we observed that cell lysates containing Sph1 accelerated the aggregation of aSyn (Fig. 5C), reinforcing our conclusions from the inclusion formation experiments and further supporting the modulatory role of Sph1 in aSyn aggregation. These findings open new avenues for targeting the Sph1 and aSyn interaction as a potential therapeutic strategy for PD and other synucleinopathies.

Furthermore, our findings also reinforce the protective role of Hsp70, which reduces inclusion size and number, suggesting that molecular chaperones dynamically regulate the aggregation landscape and that inclusion formation may be part of a broader proteostasis network. Another important aspect revealed by our model is the localization of VN-Sph1 + aSyn-VC complexes at the cell membrane, raising several questions about the role of membrane interactions in inclusion formation. It is well established that aSyn associates with various cellular membranes, such as those of synaptic vesicles, and this interaction is believed to be relevant to its physiological function^70^. While the intrinsic affinity of aSyn for lipid membranes—especially those with high curvature—is well documented, our data suggest that Sph1 further modulates this localization, potentially by affecting local membrane composition, curvature, or associated protein networks, since enrichment of inclusions near membrane compartments and vesicular trafficking hubs, especially those associated with the trans-Golgi network (TGN) and the endo-lysosomal system. However, whether membrane binding also contributes to aSyn aggregation, particularly in the context of Sph1 co-expression, remains unclear. We speculate that Sph1 synergizes with aSyn’s membrane-binding propensity to direct inclusion formation toward subcellular regions involved in membrane trafficking. This could represent a cellular strategy to compartmentalize aggregation-prone proteins, potentially as a protective mechanism to limit their interference with essential processes such as vesicle transport and protein sorting, and even release. In support of this, we observed that the co-expression of both proteins decreased the release and/or uptake of aSyn (Fig. 6), indicating that inclusion formation not only affects intracellular trafficking dynamics but may also impede aSyn propagation between cells. Notably, the sequestration of lysosomes and AP-1 vesicles into these inclusions indicates that Sph1-aSyn aggregation can physically disrupt post-Golgi trafficking pathways. This observation is consistent with previous reports of early trafficking defects in synucleinopathy models and lends further support to the hypothesis that aSyn aggregation contributes to TGN dysfunction, a recognized early pathogenic event in PD^71–73^. Further studies will be necessary to determine whether this localization is driven by alterations in membrane lipid content, fluidity, or other signaling pathways activated by Sph1. Perhaps, Sph1 may function as a sentinel or adaptor protein, capable of sensing misfolded or aggregation-prone species and guiding them into cytoprotective inclusions. This scaffolding function of Sph1 may not be limited to aSyn, as it is known to interact with other PD-related proteins such as Parkin, PINK1, LRRK2, and SIAH, hinting at a broader role in disease-associated aggregation. Thus, Sph1 may serve as a molecular hub coordinating protein aggregation and clearance, balancing between sequestration and degradation to maintain cellular homeostasis. Notably, although Sph1 expression appears to mitigate Golgi-associated aggregation in our model, its expression patterns in PD pathology remain underexplored. While our study highlights the potential of Sph1 in modulating aSyn aggregation, the expression levels of Sph1 in pathological conditions have yet to be systematically assessed. Previous studies reported that epigenetic modifications, such as hypermethylation of the *SNCAIP* gene in the cortex of PD patients, may reduce Sph1 expression in certain PD subtypes, particularly in cases with dementia. However, further studies in larger, clinically diverse cohorts are needed to determine whether altered Sph1 levels are a driver or consequence of neurodegeneration^74^.

In conclusion, our study reports not only on the development of a novel model for studying aSyn aggregation in living cells, but also provides a comprehensive analysis of the interaction between Sph1 and aSyn in a cellular context, revealing that Sph1 is a crucial modulator of aSyn assembly. Future studies will need to detail the interaction in living multicellular organism, and to investigate the role of Sph1 in other neurodegenerative diseases, as well as to explore the potential of targeting Sph1 to modulate protein aggregation and improve cellular proteostasis. However, the ability to modulate the aggregation process by altering Sph1 expression or disrupting the Sph1-aSyn interaction paves the way for testing and screening putative therapeutic strategies for PD and related synucleinopathies.

## Methods

### Sph1 BiFC

The cDNA sequence of human Sph1 was subcloned from the pcDNA3.1/V5-His-TOPO expression vector (Invitrogen, USA)^63^ into the Venus-BiFC plasmids previously described^38^. Briefly, the Sph1 sequence was cloned using specific primers 3’ of the N-terminal fragment of Venus (VN; VN-Sph1), corresponding to amino acids 1–158 (VN-fragment), and upstream of the C-terminal fragment (VC; Sph1-VC), corresponding to amino acids 159–239, using specific primers. The primers contained the restriction enzyme sites AflII at the 5’ and XhoI at the 3’-end, respectively. The sequences of the primers used were as follows:

Forward VN-Sph1: 5’-CCCCTTAAGATGGAAGCCCCTGAATACCTTG-3´

Reverse VN-Sph1: 5’-CCCCTCGAGTTATGCTGCCTTATTCTTTCCTTTGCTAGCGGAGCTGG-3´

Forward Sph1-VC: 5’-CCCCTTAAGATGGAAGCCCCTGAATACCTTG-3´

Reverse Sph1-VC: 5´-CCCCTCGAGTGCTGCCTTATTCTTTCCTTTGCTAGCGGAGCTGG-3´

The PCR fragments were digested and cloned into the aSyn BiFC constructs by replacing the aSyn insert. All constructs were verified by DNA sequencing.

To create the aSyn and Sph1 fusion, Sph1 was cloned into pcDNA3.1-aSyn-myc plasmid between Xho1 and Not1 restriction enzyme sites. aSyn-myc is inserted with Nhe1 and Xho1 restriction sites. The primers used were the follow:

Forward Sph1-xho: 5’- GATCCTCGAGGAAGCCCCTGAATACCTTGATT-3’

Reverse Sph1-not: 5’- GATCGCGGCCGCTTATGCTGCCTTATTCTTTCCTTTGC-3’

### Site directed mutagenesis on Sph1

For mutagenesis, the primers were designed according to the manufacturer’s instructions. We used the following primers:

Forward Sph1_A519N: 5’-GACCTGCATGTCGCTGAACTCTCAAGTGGTGAAG-3’

Reverse Sph1_A519N: 5’-CTTCACCACTTGAGAGTTCAGCGACATGCAGGTC-3’

Forward Sph1_K527A: 5’-GTGGTGAAGTTAACCGCGCAGCTAAAGGAAC-3’

Reverse Sph1_K527A: 5’-GTTCCTTTAGCTGCGCGGTTAACTTCACCAC-3’

Site-directed mutagenesis was performed using the QuickChange II Site-Directed Mutagenesis Kit (Agilent Technologies), following the manufacturer’s instructions. The mutagenesis was performed on the plasmids encoding the Sph1 BiFC system^38,39^ and confirmed by a DNA sequencing program.

### Cell culture

Human neuroglioma cells (H4) were maintained in Opti-MEM I Reduced Serum Medium (Life Technologies) and Human Embryonic Kidney 293 (HEK) cells were cultured in Dulbecco’s Modified Eagle Medium (DMEM, Life Technologies). Both media were supplemented with 10% Fetal Bovine Serum Gold (FBS) (PAA) and 1% Penicillin-Streptomycin (PAN), and both cells lines were maintained at 37°C in an atmosphere of 5% CO2.

Primary midbrain neuronal cultures were prepared from E18 Wistar rat embryos (mixed sex) as described previously^75^. The cells were seeded on poly-L-ornithine coverslips (Sigma-Aldrich) and cultured in a serum-free medium (Dulbecco’s Modified Eagle Medium/Nutrient Mixture F-12 [DMEM/F-12] consisting of N1 supplement, bovine serum albumin, glutamine (Sigma), and penicillin/streptomycin mixture (Gibco). Every 3 days, one-third of the medium was removed and replaced with fresh medium.

Primary mouse neurons were prepared from the hippocampus of embryonic day E16–E18 CD1 mouse embryos as previously described^36,76^. All procedures were performed according to the National Institutes of Health Guide for the Care and Use of Experimental Animals and were approved by the University of Pennsylvania Institutional Animal Care and Use Committee. Dissociated hippocampal neurons were plated at 100,000 cells/well (24-well plate) in Neurobasal medium (ThermoFisher) supplemented with B27 (ThermoFisher), 2 mM GlutaMax (ThermoFisher), and 100 U/ml penicillin/streptomycin (ThermoFisher).

### Cell transfection

The day before transfection, 120,000 cells were plated in 12-well plates or in microscope glass bottom plates (iBidi) dishes, or 240,000 cells in 6-well plates (Corning). The cells were transfected with equimolar amounts of the plasmids (or otherwise indicated in the figure) using Rotifect Plus (Carl Roth) or Metafectene (Biotex), according to the manufacturer’s instructions. After 24 or 48 h the media and the cells were either collected or stained for further analysis. For the competing assay, aSyn BiFC was first expressed, and after 24 h, either empty vector, VN-Sph1 or Sph1-VC was expressed.

Twenty-four hours prior to transfection, the 80,000 cells were plated in a 12-well plates (Costar, Corning). An equal amount of the plasmids encoding aSyn and WT or mutant Sph1 were transfected using FuGENE6 Transfection Reagent (Promega) in a ratio of 1∶3 according to the manufacturer’s instructions. Forty-eight hours after the transfection, the media were collected and frozen for further experiments. Additional cells were subjected to immunocytochemistry for studying aSyn inclusions.

Neurons were transfected with Lipofectamine300 (ThermoFisher) at DIV 4 with equimolar amounts of the plasmids, following the manufacturer’s recommendation. The cells were cultured until DIV 6, after which the cells were fixed and stained.

Hippocampal neurons were transfected with calcium-phosphate method at DIV 4 as previously described^77^. Briefly, 2× BES-buffered saline solution containing phosphate ions (50 mM BES, 280 mM NaCl, 1.5 mM Na_2_HPO_4_xH_2_O, pH 7.02) were mixed with calcium chloride solution (2.5 M) containing equimolar amounts of the plasmids. The cell growth media was collected and reserved, and OPTIMEM without adds where added to the cells. Cells were incubated with plasmid-calcium-phosphate coprecipitates for 40 min followed by media change (growth media previous collected). The cells were fixed and stained at DIV 6.To perform the co-cultures, 24 h post-transfection, cells were washed once with 1x PBS. Following trypsinization, 50,000 cells per condition were plated into new wells. At designated time points (24, 48, and 72 h), cells were fixed, and the number of Venus-positive cells were counted. Additionally, the culture media from HEK 293 cells were collected and stored at −80 °C for subsequent ELISA experiments.

### Immunocytochemistry

After transfection, HEK, H4 cells, primary cortical or hippocampal neurons were fixed with 4% paraformaldehyde at room temperature (RT) for 10 minutes. After washing 3 times with 1xPBS, the cells were permeabilized for 20 min with 0.1% Triton X-100 (Sigma-Aldrich)/1xPBS. Then, the cells were blocked in 1.5% normal goat serum (PAA)/1xPBS for 1 h and incubated with primary antibody. The primary antibodies used in this study were: anti-aSyn (610787, BD Biosciences), anti-Sph1 (sc-365741, Santa Cruz), anti-Giantin (ab80864, Abcam), anti-MAP2 (17490-1-AP, Proteintech) overnight (ON), and secondary antibody (Alexa Fluor 568 donkey anti-mouse IgG or Alexa Fluor 568 goat anti-rabbit IgG, (Life Technologies- ThermoFisher Scientific) incubated for 2 h at RT. Cells were stained with Hoechst 33258 (Life Technologies- Thermo Fisher Scientific) (1:5000 in DPBS) for 5 min, and the coverslips mounted for imaging.

For the organelle studies, HEK 293 T were fixed and blocked as previously described, and the following primary antibodies were used: anti-Giantin (ab37266, Abcam), anti-PMP70 (SAB4200181, Sigma), anti-AP-1 (610385, BD Transduction Laboratories), anti-TOM20 (sc-11415, Santa Cruz Biotech), anti-G3BP1 (WH0010146M1, Sigma), anti-DCP1B (13233, Cell Signaling Technology), anti-CKAP4 (AB_1731083), anti-LAMP1 (sc20011, Santa Cruz Biotechnology), anti-GAPDH (sc-47724, Santa Cruz Biotechnology). For immunofluorescence, we used the following secondary antibodies: anti-rabbit IgG Cy3-conjugated (Sigma-Aldrich C2306), anti-mouse IgG Cy3-conjugated (Sigma-Aldrich C2181), anti-rabbit IgG Cy5 conjugated (Invitrogen A10523), anti-Mouse IgG H&L (Alexa Fluor 488) (Abcam).

### Quantification of VN-Sph1 + aSyn-VC and SynT inclusions

At least fifty positively transfected cells *per* condition were quantified based on the presence or absence of inclusions. For the SynT + Sph1 models the transfected cells were detected and scored based on the aSyn inclusion pattern and classified into four groups: cells without inclusions, less than five inclusions ( < 5 inclusions), between five to nine inclusions ( ≥ 5–9 inclusions) and more than ten inclusions ( ≥ 10 inclusions). All the results were expressed as the percentage of the total number of transfected cells obtained from three independent experiments.

For the quantifications of the different VN-Sph1 + aSyn-VC ratios, at least 300 cells *per* condition were used.

### FRAP analyses

The HEK cells were plated in iBidi dishes and transfected as mentioned previously. FRAP experiments were performed on a Leica 6000B microscope equipped with an incubator to maintain the 5% CO_2_ and 37°C, and with UGA-42 Firefly scanner-based systems (Rapp OptoElectronic, Germany).

The samples were initially imaged for three consecutive time-frames (pre-bleach- I_0_), and then a circular region of interest (ROI) was bleached with three iterations scan (30% of 405 nm laser power) for 227 ms. A maximum of three inclusions per cell was bleached/recorded, and fluorescence recovery was measured every 20 s for 5 min.

To quantify inclusion intensity recovery over time, we used a customized script for Fiji (ImageJ) software (Wayne Rasband, NIH, Bethesda, MD, USA). Fluorescence intensities of the defined ROIs were measured, and the average intensities pre-bleach and after bleach were corrected according to the background. The average fluorescence in the bleached area I(t) at each time point t was used to normalize FRAP recovery curves.

1. $${FRAP\; recovery\; curve}=\frac{I(t)}{{I}_{0}}$$

All nonlinear curve fitting and the statistical comparisons were performed using GraphPad Prism 4.0.

### Live cell imaging microscopy

For live cell imaging, 4-well iBidi or Cellview cell culture dish (Greiner Bio One) were used. Alternatively, cells were grown on glass slides (Marienfeld). Confocal images and movies were acquired using SP8 (Leica) confocal microscope equipped with a temperature and CO_2_ incubator, using a 60x PlanApo VC oil objective NA 1.40, and 406 nm, 488 nm, 561 nm, and 640 nm lasers. Image processing was performed using Fiji (ImageJ) software.

### Particle size analyses

The images were analyzed using a customized script in Fiji (ImageJ) software. Briefly, calibrated images (with pixels converted to micrometers) were used to automatically detect the particles. First, Otsu threshold was applied to convert the image to a binary image (black and white). Then, the minimum and maximum area sizes were set to exclude anything that is not the object of interest in the image. Once all the parameters were setup, the particle analysis was performed automatically. The data was blotted in a graph using BIN, which was performed in GraphPad Prism 7.0.

### Oxidative stress assessment

48 h after transfection, cells were treated with 150 μM sodium arsenite for 2 h followed by lysis and collection for WB analysis.

### Western blot analyses

Proteins were extracted using 1xPBS pH 7.4 with Protease Inhibitor Cocktail (1 tablet/10 mL) (Roche Diagnostics) and sonicated during 10 s at 10% power. Protein concentration was determined by Bradford assay (BioRad Laboratories), using an Infinite M200 PRO plate reader (Tecan, Lta). The sample were mixed with 5x Laemmli buffer (250 mM Tris pH 6.8, 10% SDS, 1.25% Bromophenol Blue, 5% β-Mercaptoethanol, 50% Glycerol), and denatured for 5 min at 95 °C. Precast gels were loaded with 30 μg protein, and the samples were separated on 4–20% or 4–15% Mini-PROTEAN TGX Precast Protein Gels (BioRad Laboratories). The gel was transferred to a PVDF membrane using the iBlot transfer system (ThermoFisher), according to the manufacturer’s instructions. Membranes were blocked with 3% (w/v) BSA (NZYTech), 1xTris Base Solution/0.05% Tween (TBST), followed by the primary antibodies diluted in 3% BSA/TBST ON at 4 °C. The following antibodies were used: anti-aSyn (610787, BD Biosciences), anti-Sph1 (sc-365741, Santa Cruz), and anti-beta-actin (A2228, Sigma-Aldrich). After washing, the membranes were incubated for 2 h with anti-mouse IgG horseradish peroxidase labeled secondary antibody (GE Healthcare). Proteins were detected by ECL chemiluminescent detection system (Millipore) in Fusion FX (Vilber Lourmat).

### Native PAGE

48 h after transfection, HEK cells were lysed in 1xPBS pH 7.4 containing Protease Inhibitor Cocktail tablet (1 tablet/10 mL) (Roche Diagnostics) and separated in 4–16% gradient Native pre-cast gel (SERVA Electrophoresis GmbH). Gels were run according to the manufacturer’s instructions and transferred as previously described. The following antibodies used were: Syn1 for total aSyn (610787, BD Biosciences), MJFR-14-6-4-2- conformation specific (ab209538, Abcam) and 5G4- aggregated aSyn (MABN389, Merck).

### Proteinase K resistance

Cells were collected 48 hours after transfection in 1xPBS pH 7.4 with Protease and Phosphatase Inhibitor Cocktail (Roche Diagnostics). The lysates were sonicated for 10 s at 40–50% power, and then incubated with 2.5 μg/ml freshly prepared PK for different time periods (0, 5, 10 and 15 min). The digestion was stopped by adding the Laemmli buffer. The lysates were boiled at 95 °C for 5 min, loaded onto 4–20% Mini-PROTEAN TGX Precast Protein Gels (BioRad Laboratories), and immunoblotted as mentioned above. The antibody used was Syn1 (610787, BD Biosciences).

### Cell viability

To test the cytotoxicity, adenylate kinase release into cell media was measured 24 h after transfection of HEK cells, using ToxiLight bioassay kit (Lonza) according to the manufacture’s recommendations.

For the lactate dehydrogenase (LDH) cytotoxicity assay (Roche Diagnostics, Mannheim, Germany) (supplementary data Fig. 5), the reaction mixture was prepared according to the manufacturer. The growth media from HEK cells were plated in triplicates in a 96-well plate in a ratio of 1∶1 with the reaction mixture. The absorbance measurements were performed in a TECAN Infinite 200 Pro plate reader at 490 nm. To determine the percentage cytotoxicity, the average absorbance values were subtracted with the average absorbance value obtained in the background control. The percentage of toxicity was calculated as indicated by the manufacturer.

The cell proliferation was evaluated after 24 h of transfection using the AlamarBlue proliferation assay (BioRad Laboratories, USA). AlamarBlue was added at 1:10 ratio to the cells and quadruplicates of each condition was measured every hour on the infinite M200 fluorescence plate reader set to 37 °C (Tecan Systems) with excitation 570 nm, emission 600 nm. The percentage reduction of AlamarBlue was calculated using the following formula:

(2) $$\frac{\left(O2\times A1\right)-(O1\times A2)}{\left(R1\times N2\right)-(R2-N1)}\times 100$$

O1= Molar extinction coefficient of oxidized AlamarBlue at 570 nm

O2 = E of oxidized AlamarBlue at 600 nm

R1 = E of reduced AlamarBlue at 570 nm

R2 = E of reduced AlamarBlue at 600 nm

N1 = absorbance of negative control well (media with AlamarBlue but no cells) 570 nm

N2 = absorbance of negative control well (media with AlamarBlue but no cells) 600 nm

48 h after transfection, the HEK cells were trypsinized and cell viability was quantified using an automated cell counter (Countess from Invitrogen) at a 1:1 ratio of cells to trypan blue.

### Size exclusion chromatography and dot blot

Cells were harvested 48 h after transfection in phosphate buffer (TBS with 0.5% Triton X-100), freshly supplemented with protease inhibitor cocktail, and centrifuged at 5 °C and 10,000 × *g* for 10 min. 1.5 mg of total protein, in a maximum volume of 500 µl, was filtered using a 0.45 µm spin-X centrifuge filter before loading onto a Superose 6 column (Superose 6 10/300GL, GE Healthcare, Sweden) and subsequently processed by SEC-HPLC (Äkta Purifier 10, GE Healthcare, Sweden). Each SEC-HPLC run was performed at a flow rate of 0.5 ml/min for 1.2 column volumes in ammonium acetate buffer (50 mM, pH 7.4). After the SEC-HPLC run, the collected fractions were boiled at 95 °C for 5 min, centrifuged at 14,000 × *g* for 5 min, and loaded into a nitrocellulose membrane using a dot-blot apparatus. The immuno-signal was detected as described above, using the Syn1 antibody to detect total aSyn.

### Quantification of Golgi fragmentation

The Golgi morphology was assessed and scored into three groups as we previously published^39,44,45^ (normal, diffused, and fragmented). Briefly, 48 h after transfection, HEK cells were fixed and stained for Golgi apparatus as described in the previous section. For our analyses we discarded cells that were in metaphase (visible by the nuclear morphology). The morphology of the Golgi apparatus was assessed taking into consideration its shape and the area/distance to the nucleus: normal- compact/close to the nucleus; diffused: disperse/close to the nucleus; fragmented: dispersed throughout the cell. Three independent experiments were performed to determine the Golgi morphology of the cells expressing Sph1 and/or aSyn and the surrounding cells.

### Cell cycle arrest

For the double-thymidine cell cycle arrest, the cells were initially grown in medium containing 2 mM thymidine for 16 h and then rinsed in 1xPBS, and maintained in growth medium for 8 h. During this recovery period, the cells were transfected as before. Following this, the cells were exposed again to thymidine for an additional 16 h before the final release of the cell cycle arrest. After 12 h, the cells were rinsed, fixed, and prepared for analysis.

### Measurements of the area of the Golgi apparatus

To measure the Golgi area, we first converted our images (the same ones used for quantifying Golgi fragmentation) to 8 bits. Subsequently, we drew a box around the region of interest (the Golgi apparatus) and employed the Hull and Circle plugin from ImageJ to obtain classical shape descriptors, like circularity, convex area, convex perimeter, bounding box width, and height (as shown in supplementary Figure 2C). The convex area was chosen as the representative data, closely resembling the Golgi staining area distributed throughout the cytoplasm. These data were then plotted in a frequency distribution graph, in which we used relative frequencies to determine the percentage of values in each bin.

### Quantification of aSyn levels in the cell culture medium

For the BiFC model, a 96-well high-binding polystyrene microtiter plate (Corning, USA) was coated with 50 ng per well of Syn1 (610787, BD) antibody for the detection of aSyn and incubated at RT overnight. Following incubation, the wells were washed three to five times with washing buffer (6.49 mM NaH₂PO₄·H₂O, 43.5 mM Na₂HPO₄·2H₂O, 3 M NaCl, 2% Tween 20, 0.15% Kathon). Cell media was added to the wells and incubated at RT with shaking for 2 h. After incubation, the wells were washed three times with washing buffer. Subsequently, a primary antibody targeting αβγ-synuclein (Santa Cruz Biotechnology, FL-140, USA) was diluted 1:200 in incubation buffer (1x PBS, 0.1% Tween 20, 1% BSA) and incubated for 1 h at RT with shaking to facilitate binding of the primary antibody. Following primary antibody incubation, the wells were washed five times with washing buffer. Next, an anti-rabbit horseradish peroxidase (HRP)-conjugated secondary antibody (GE Healthcare, Finland) was diluted 1:5000 in incubation buffer, and incubated for 1 hour with shaking, after which the wells were washed five times with washing buffer. The HRP activity was detected by adding the K-blue aqueous substrate (TMB), which was incubated for 20 min to allow color development. The reaction was subsequently stopped by the addition of 1 M sulfuric acid (H₂SO₄), halting further enzymatic activity. The absorbance was measured at 450 nm using a Tecan Infinite M200 plate reader.

The detailed protocol for the aggregation model has been previously published. Briefly, 96-well Multi-Array standard plates (Meso Scale Discovery, Gaithersburg) were coated with 30 µl of MJF1 clone 12.1 (generously provided by Liyu Wu, Epitomics, Burlingame) as capture antibodies at 3 µg/ml in PBS. The plates were incubated overnight at 4°C without shaking. Following incubation, plates were washed three times with 150 µl PBS-T (PBS supplemented with 0.05% Tween-20). After washing the unbound capture antibodies, the plates were blocked with 1% BSA/PBS-T for 1 h at RT with shaking at 300 rpm. Standards and samples collected from HEK and H4 cells were diluted in 1% BSA/PBS-T and 25 µl volumes were added to the wells, followed by incubation 1 h at RT with shaking at 700 rpm. Plates were washed again and 25 µl Sulfo-TAG labeled ASYN (BD Biosciences) were applied at 1 µg/ml. After a final washing 150 µl 2x Read Buffer T (MSD) were added, and plates were read using the Sector Imager 6000 (MSD).

### RT-QuIC measurement of aSyn seeding activity

The aSyn RT-QuIC analysis was conducted as described previously^78^, with minor modifications. The preparation of recombinant aSyn monomer and Pre-formed fibrils (PFFs) was done as previously described^79^. Briefly, the RT-QuIC reaction mix was composed of 100 mmol of PIPES buffer (pH 6.5, 0.5 M Fisher), 170 mmol of sodium chloride, 0.1 mg/ml of recombinant human WT aSyn (filtered through a 100 kDa filter immediately prior to use). 20 μmol of thioflavin T (ThT). Each reaction consisted of 2 μL of WT human PFFs (0.007 μg/μL) or cell lysate (Bradford was performed, as both sample presented with comparable protein amounts) was used as a seed and 98 μL of aSyn RT-QuIC reaction mixture with three 0.8 mm silica beads (OPS Diagnostics, Lebanon, NJ) per well in a 96-well plate (Thermo, Nalgene Nunc International, 265301). The plates were closed with a plate sealer film (ThermoFisher) and incubated at 37 °C in a BMG FLUO star Omega plate reader. The incubation plates were subjected to cycles of 30 s shaking (400 rpm double orbital) and 30 s rest for 60 h. ThT fluorescence measurements (450 ± 10 nm excitation and 480 ± 10 nm emission; bottom read) were taken every 30 min. Each sample was tested in triplicate, and all the positive samples were amplified in all three of their technical replicates.

### Statistical analyses

Data were analyzed using GraphPad Prism 4 (San Diego California, USA) software. *P* values were calculated using a two-tailed Student *t-test* unless otherwise specified.

For the analysis of organelles colocalization, three or more independent experiments were performed to obtain the data. *P* values were calculated by two-tailed Student *t-test*, or one-way ANOVA for samples following normal distribution determined by the Shapiro-Wilks test. The equality of variances was verified by Brown-Forsythe or F test. Mann-Whitney (2 groups) or Kruskal-Wallis (multiple groups) tests were used for samples that did not follow a normal distribution. The sample sizes were not predetermined.

To analyze the expression levels of aSyn and Sph1 in relation to the modulation of VN-Sph1 + aSyn-VC inclusions, *P* values were calculated using a two-way ANOVA after confirming normal distribution of the data with the Shapiro–Wilk test.

Statistical significance was assessed where * corresponds to p < 0.05, ** corresponds to p < 0.01, *** corresponds to p < 0.001 and **** corresponds to p < 0.0001.

## Supplementary information

Supplementary Information

Supplementary movie 1

Supplementary movie 2

Supplementary movie 3

## Data Availability

All data are available within the Article and Supplementary Files, or available from the corresponding authors upon reasonable requests.
