# Supplementary material for: Synphilin-1 modulates alpha-synuclein assembly, release and uptake
Source: NPJ Parkinsons Dis. 2025 Nov 20;11:326. doi: 10.1038/s41531-025-01144-3 (PMC12635182; doi:10.1038/s41531-025-01144-3)
Supplement: Supplementary file 1 — Supplementary Information [file 41531_2025_1144_MOESM1_ESM.pdf]

Supplementary data figure 1

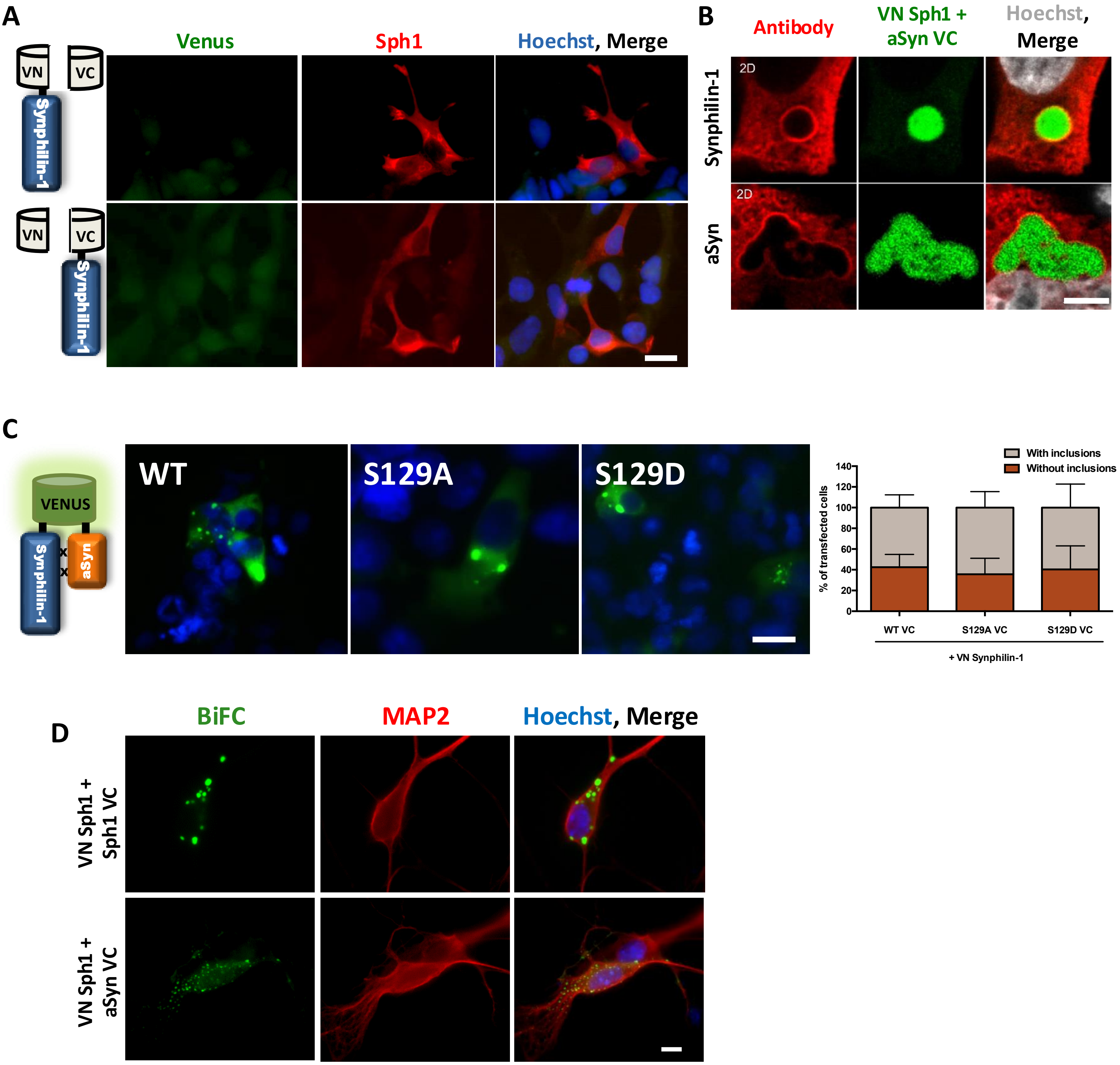

Supplementary Figure 1. A. BiFC controls. No positive signal was observed in cells transfected with either VN-Sph1 + -VC or VN- + Sph1-VC, confirming the specificity of the interaction between Sph1 and aSyn. **B. Confocal Imaging of VN-Sph1 + aSyn-VC Inclusions.** High-resolution confocal images provide a detailed view of VN-Sph1 + aSyn-VC inclusions in cells, revealing their morphology (scale bar: 5 μm). **C. Effect of phosphorylation on inclusion formation.** The formation of VN-Sph1 + aSyn-VC inclusions remains unchanged regardless of whether aSyn phosphorylation at S129 is blocked or promoted, indicating that phosphorylation at this site does not significantly affect the interaction of both proteins. **D. VN-Sph1 + aSyn-VC inclusions in primary hippocampal.** Primary hippocampal neurons transfected at DIV4 exhibit similar VN-Sph1 + aSyn-VC inclusions to those observed in HEK 293 cells and primary cortical neurons at DIV6, demonstrating that inclusion formation is consistent across different cell types (scale bar: 30 μm).

Supplementary data figure 2

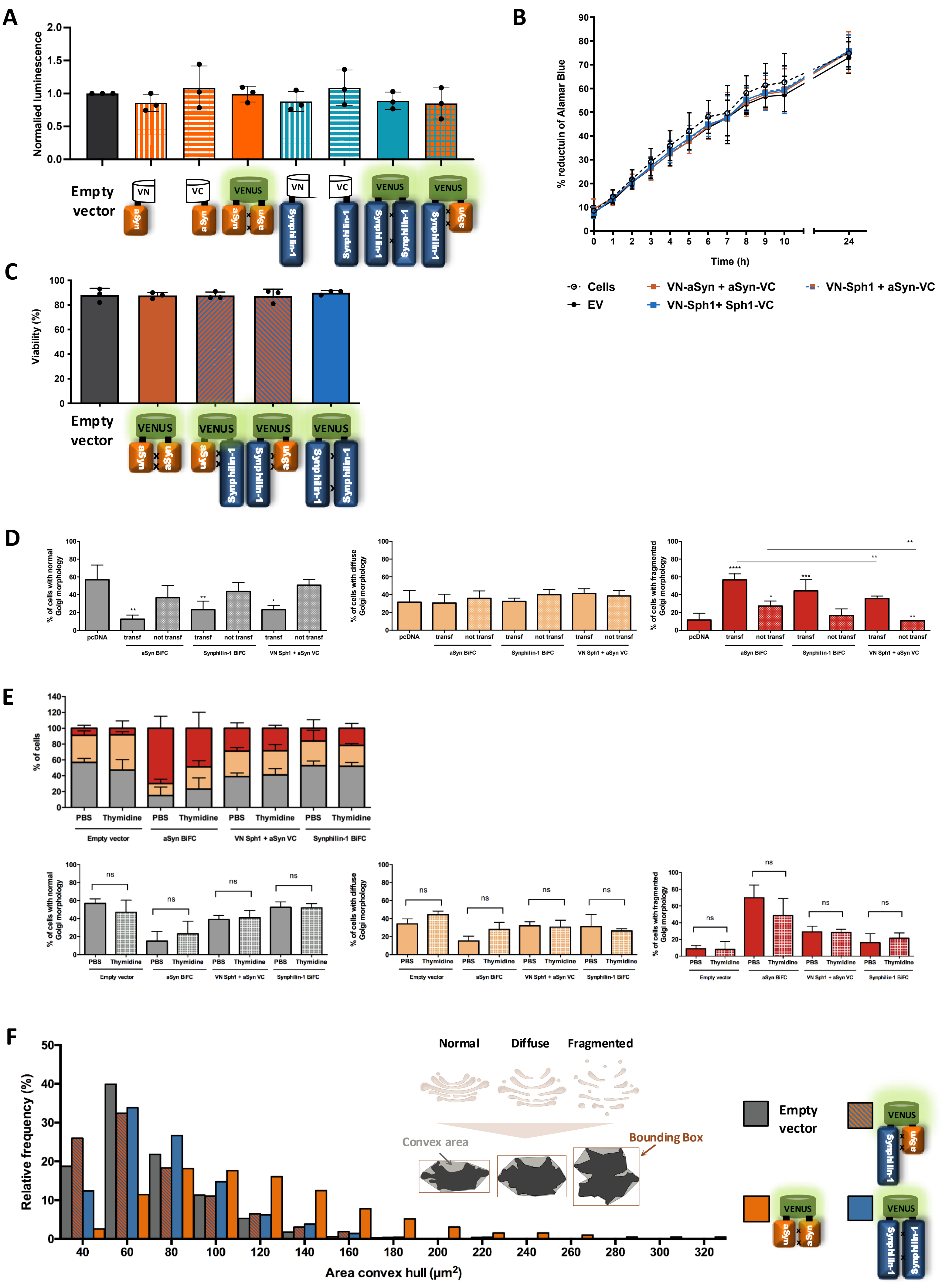

Supplementary Figure 2. Sph1 rescues the detrimental effects of aSyn on the Golgi apparatus. A-C. VN-Sph1 + aSyn-VC inclusions do not induce cytotoxicity. Cytotoxicity was assessed by measuring AK activity in the culture medium (A). No significant AK release was observed across different conditions, indicating that VN-Sph1 + aSyn-VC inclusions do not induce cytotoxicity. **B. Metabolic activity quantification.** The metabolic activity of cells was measured to assess cell proliferation under different conditions. No major differences were observed, suggesting that VN-Sph1 + aSyn-VC inclusions do not impact cell proliferation. **C. Evaluation of cell membrane integrity.** Cell viability was assessed using trypan blue exclusion, which no significant differences in viability or cell count observed across conditions were detected. All measurements were taken 24 hours after the expression of Sph1 and aSyn. n=3 **D. Assessment of Golgi morphology.** Golgi morphology was categorized as normal (gray), diffuse (yellow), and fragmented (red). aSyn BiFC significantly increased Golgi fragmentation, but this effect was counterbalanced with Sph1 expression, demonstrating the protective role of Sph1. **E. Cell cycle arrest.** Thymidine-induced cell cycle arrest at the G1/S boundary did not exacerbate aSyn's toxic effects on the Golgi morphology. No differences were observed between unsynchronized cells and those arrested in the cell cycle. **F. Distribution of Golgi area**. The Golgi area occupied by aSyn BiFC was larger compared to VN-Sph1 + aSyn-VC and Sph1 BiFC, consistent with the observed quantifications and suggesting that Sph1 can counterbalance aSyn-induced Golgi fragmentation.

Supplementary data figure 3

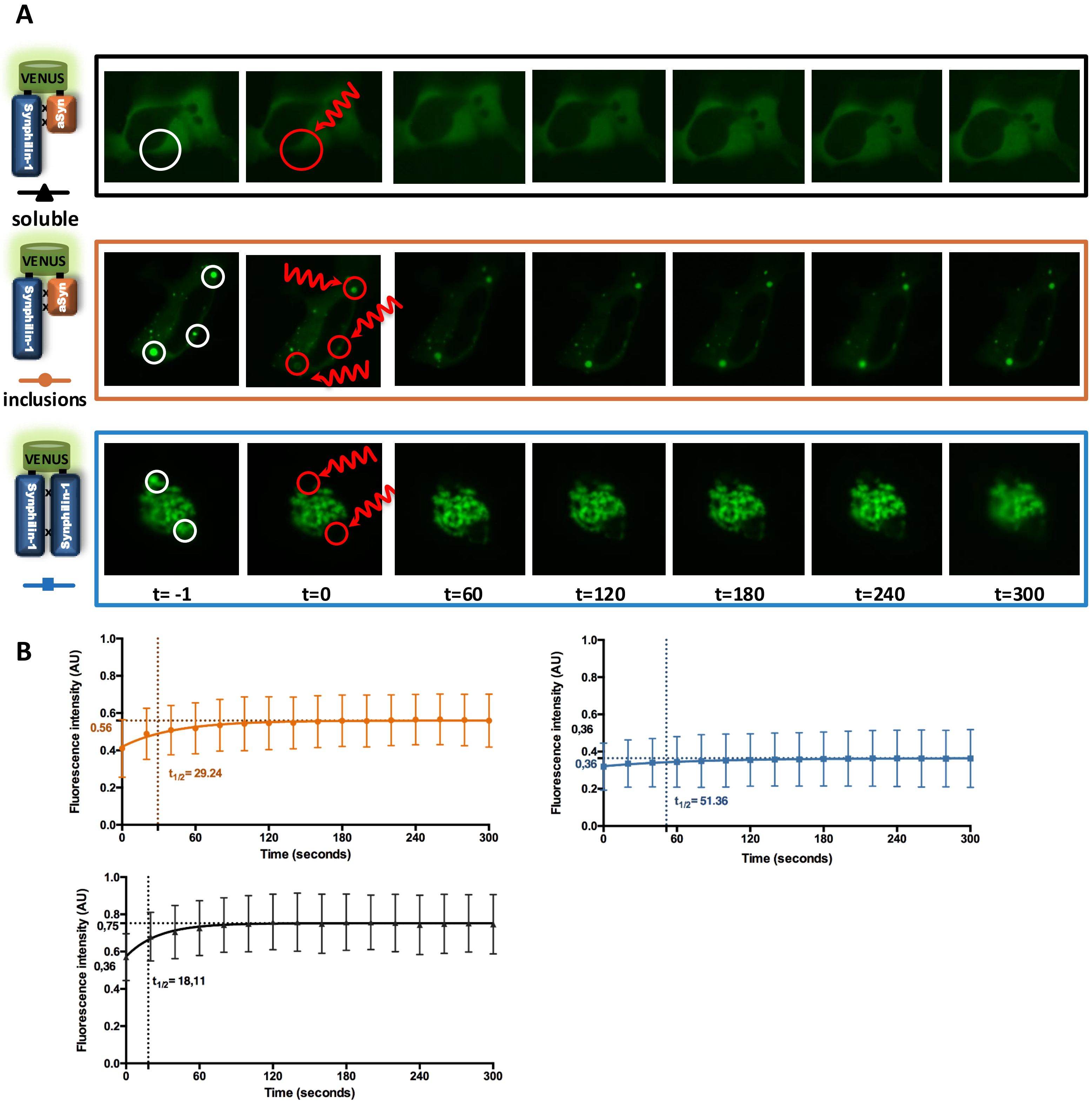

Supplementary Figure 3. Quantification of FRAP experiments. A-B. FRAP analysis. The recovery of the Venus fluorescence signal was monitored over 5 minutes after the photobleaching. Each condition was plotted in individual graphs, allowing for the visualization of fluorescence recovery kinetics. The half-time values for fluorescence recovery were calculated for each condition, providing a quantitative measure of the dynamic properties of VN-Sph1 + aSyn-VC and Sph1 inclusions (B).

Supplementary data figure 4

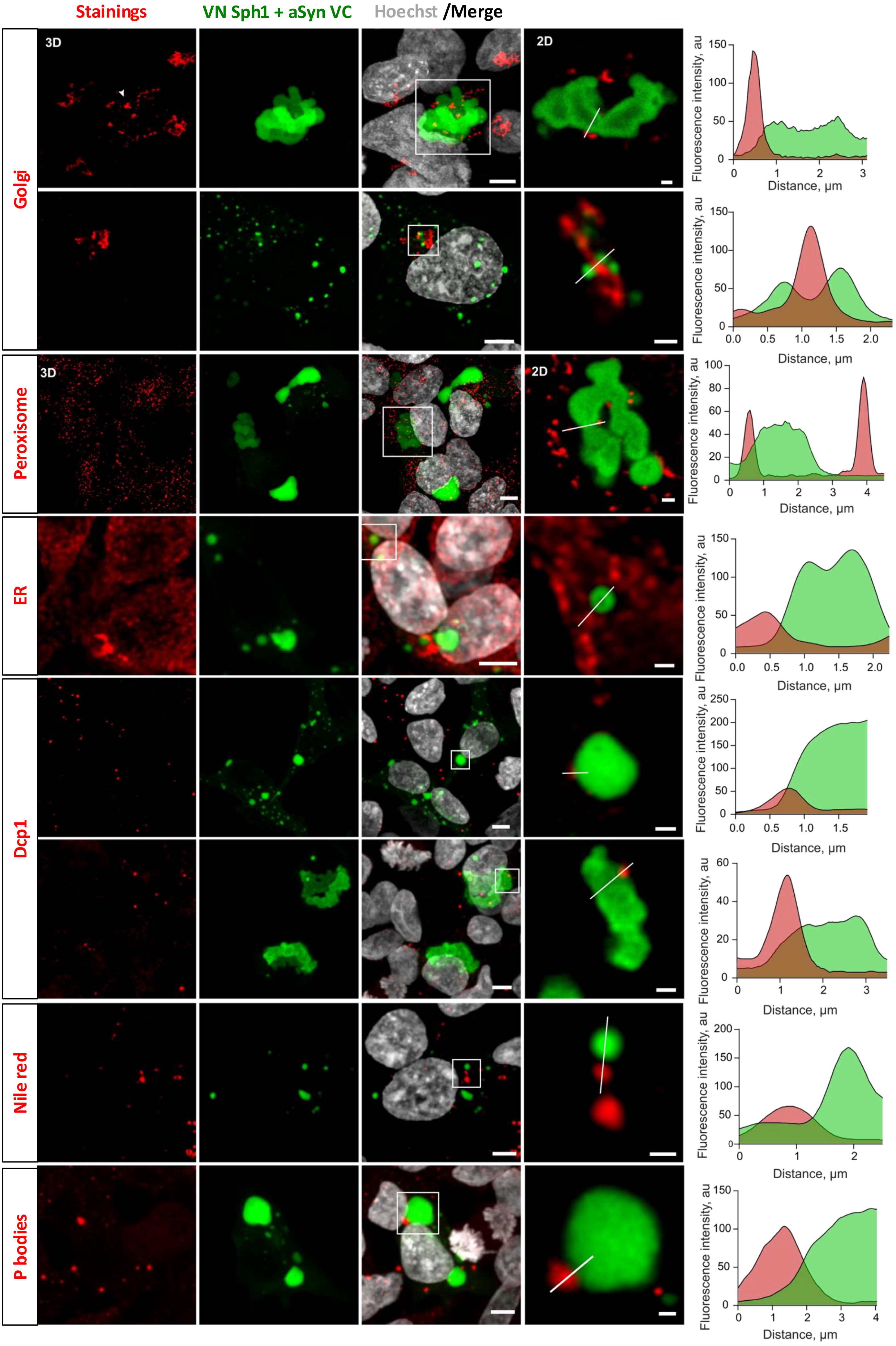

Supplementary Figure 4. Organelle imaging. Confocal imaging was used to investigate the association of VN-Sph1 + aSyn-VC inclusions with various membrane-bound and non-membrane-bound organelles. The results showed that VN-Sph1 + aSyn-VC inclusions did not co-localize with peroxisomes, ER, endoplasmic reticulum, lipid droplets, or P-bodies. Representative fluorescence intensity profiles through the inclusions (white line on the inset) are shown. Scale bar of overall image: 5 μm and crop image: 1 μm.

Supplementary data figure 5

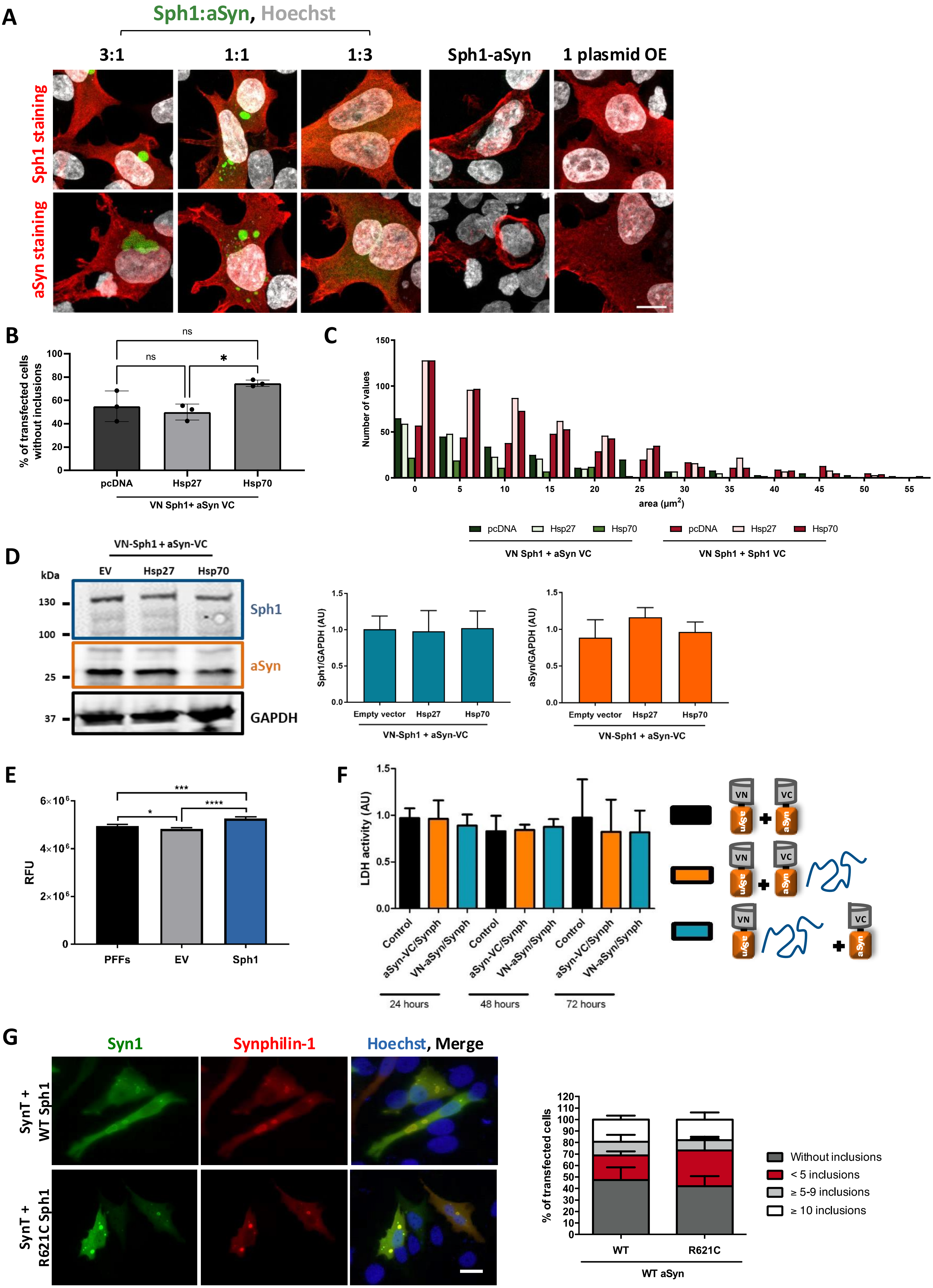

Supplementary Figure 5. VN-Sph1 + aSyn-VC localization, cytotoxicity and inclusion formation. A. VN-Sph1 + aSyn-VC localize with membranes. A plasmid was constructed to fuse Sph1 and aSyn, ensuring similar expression levels for both proteins. Confocal imaging confirmed that VN-Sph1 + aSyn-VC localize to the membrane, and this localization is independent of the expression ratio of Sph1 and aSyn. The interaction between Sph1 and aSyn is essential for their membrane localization. Scale bar: 5 μm. **B-D. Hsp70 disaggregates VN-Sph1 + aSyn-VC inclusions.** The expression of Hsp70 leads not only to a reduction in number but also to a reduction in the size of VN-Sph1 + aSyn-VC inclusions. The chaperone-mediated changes in inclusion formation occur without alterations in total Sph1 or aSyn protein levels. n=2 (D). **E. Sph1 accelerates aSyn aggregation.** The AUC shows that not only does the cell environment influence aSyn aggregation, but Sph1 also accelerates the aggregation. **F. Cytotoxicity of assessment.** The cytotoxicity of various conditions was evaluated using medium collected from H4 cells. After measuring aSyn release *via* ELISA, the same medium was analyzed for lactate dehydrogenase (LDH) release, a marker of cell damage. The percentage of cytotoxicity was calculated for each condition, with no significant differences observed after 72 hours of treatment. **G. WT and mutant Sph1 induce aSyn inclusion in H4 cells.** Both WT and mutant Sph1 induced similar number of aSyn inclusion in human H4 cells. The inclusions were quantified, with >50 cells scored per experiment and classified into different groups according to the inclusion patterns observed. n=3.Scale bar: 10 μm.

Supplementary Movie 3B upper. Illustration of the dynamics of Sph1-aSyn inclusions, highlighting the fusion of small or single inclusions into larger aggregates, indicated by arrowheads. This visualization provides insight into the aggregation process of Sph1-aSyn within the cellular environment, emphasizing the dynamic interactions between smaller inclusions.

Supplementary Movie 3B lower. The time-lapse sequence shows the reduced mobility of tubular Sph1-aSyn inclusions compared to smaller or individual inclusions. This video captures the distinctive behavior of the tubular Sph1-aSyn inclusions, highlighting their distinct characteristics.

Supplementary Movie 3C. Cells treated with cycloheximide exhibit increased fluidity of Sph1-aSyn inclusions, enabling fusion and fission events. This movie highlights the dynamic nature of the inclusions and illustrates how their behavior can be modulated, providing insights into the mechanisms driving inclusion formation and dynamics.
